# Arpra: An Arbitrary Precision Range Analysis Library

**DOI:** 10.3389/fninf.2021.632729

**Published:** 2021-06-25

**Authors:** James Paul Turner, Thomas Nowotny

**Affiliations:** School of Engineering and Informatics, University of Sussex, Brighton, United Kingdom

**Keywords:** interval arithmetic, affine arithmetic, range analysis, floating-point, reproducibility, numerical integration, spiking neural networks

## Abstract

Motivated by the challenge of investigating the reproducibility of spiking neural network simulations, we have developed the Arpra library: an open source C library for arbitrary precision range analysis based on the mixed Interval Arithmetic (IA)/Affine Arithmetic (AA) method. Arpra builds on this method by implementing a novel mixed trimmed IA/AA, in which the error terms of AA ranges are minimised using information from IA ranges. Overhead rounding error is minimised by computing intermediate values as extended precision variables using the MPFR library. This optimisation is most useful in cases where the ratio of overhead error to range width is high. Three novel affine term reduction strategies improve memory efficiency by merging affine terms of lesser significance. We also investigate the viability of using mixed trimmed IA/AA and other AA methods for studying reproducibility in unstable spiking neural network simulations.

## 1. Introduction

Computer simulations are a valuable tool for understanding the behaviour of complex natural systems, in particular in the context of Neuroscience and neural network simulations. They help us form hypotheses and so reduce the need for expensive or at times impossible experiments. Executing simulations of computational models on computers, however, means accepting a small amount of imprecision in the results stemming from rounding errors when performing floating-point arithmetic, truncation errors when using approximate numerical methods, and even errors due to limited precision representations of input data. Although small, when accumulated over time, these errors can influence the overall behaviour of a computation, sometimes with dramatic effect. In a famous example, gradual accumulation of unchecked rounding errors between January 1982 to November 1983 was causing stocks at the Vancouver stock exchange to loose around 25 points per month (Huckle and Neckel, 2019). Such events have spurred a lot of interest in analysing how numerical errors propagate through computer programs. This is particularly relevant where numerical simulations rest on limited precision floating-point arithmetic (IEEE, 1985, 2008, 2019) and errors accumulate due to the iterative nature of the numerical integration of models. To further complicate matters, there are sometimes subtle differences in the way floating-point arithmetic is implemented across architectures, and additional differences in the way compilers optimise floating-point arithmetic code (Monniaux, 2008; Whitehead and Fit-florea, 2011). The use of massively parallel General Purpose Graphics Processor Unit (GPGPU) code has further compounded the problem as a large number of FPUs are used simultaneously, all operating on the same data, with no guarantee on the order in which they will begin or finish an operation.

In this work we present the Arpra library (Turner, 2019), which uses floating-point error bounding techniques to analyse numerical error propagation in computations. We evaluate the effectiveness of a novel mixed trimmed IA/AA method against existing IA, AA and mixed IA/AA methods in example problems, including a prototype spiking neural network.

### 1.1. Background

Real numbers are in the vast majority of systems represented in floating-point number format *f* = *s* · *m* · *b*^*e*^, with *s* ∈ {−1, 1} and *m* ∈ [1, *b*), where *s*, *m* and *e* are, respectively the sign, significand (also known as the mantissa) and exponent of *f*, and *b* is the base of the floating-point system (usually two). The IEEE-754-1985 standard (IEEE, 1985), and later revisions (IEEE, 2008, 2019), define the 32 bit single-precision and 64 bit double-precision floating-point formats, corresponding to the float and double types in C-like programming languages. The standard dictates that a subset of floating-point functions must be correctly rounded, meaning the result must be computed as if in infinite precision and then rounded to a nearby floating-point number according to the selected rounding mode.

#### 1.1.1. Numerical Errors

Because this representation of real numbers is discrete and approximate, small rounding errors can be introduced whenever an FPU and math library are utilised. There are three commonly used measures of numerical error (Goldberg, 1991), the first two are absolute error, error_abs_(*f, r*) = |*f* − *r*|, and relative error, ${\text{error}}_{\text{rel}}\left(f,r\right)=|\frac{f-r}{r}|$, where *f* is the computed floating-point value, and *r* is the exact value. Thirdly there is the error in terms of units in last place (ULP) of the significand, ${\text{error}}_{\text{ULP}}\left(f,r\right)=|m-\frac{r}{b^{e}}|b^{p-1}=\frac{\left|f-r\right|}{b^{e}}b^{p-1}\in \left[0,b^{p}\right)$, where *p* is the precision of *f* (the number of digits in *m*) and *e* is the exponent of *r*.

While rounding errors occur only in the least significant digit, they can rapidly be amplified, for instance through a process called catastrophic cancellation. This occurs if two approximately equal floating-point numbers are subtracted from each other so the least significant bits of the two numbers determine the most significant bits of the result. For a detailed exploration of rounding error analysis, and strategies of minimising errors based on specific ordering of arithmetic operations, see Higham (2002). For a complete primer for floating-point arithmetic in general, we refer you to Muller et al. (2018).

Another type of error occurs when numerically integrating ordinary differential equations. This involves the discretisation of the equations into difference equations with a finite time step δ*t* and limited order of approximation, causing so-called truncation errors. When numerically integrating a model on a computer, the overall error is a combination of rounding error from the basic computations, and truncation error from the integration method. There is a trade-off between reducing truncation error through smaller time steps and increased rounding errors due to more ensuing floating-point operations (Chesneaux et al., 2009).

#### 1.1.2. Reproducibility

A serious repercussion of the existence of unavoidable numerical errors is the lack of reproducibility in numerical simulations. In the context of serial (single threaded) execution, this can be a problem when compilers and CPU architectures comply to different standards (Monniaux, 2008; Whitehead and Fit-florea, 2011). For instance, in IEEE-754-1985, the only floating-point operations that are required to be correctly rounded are the basic (+,−,/,*,√) operations and floating-point conversion functions. However, in later IEEE-754 revisions, the ‘fused multiply-add’ (FMA) function is included in this list. FMA instructions are now shipped as standard with AMD and Intel processors, respectively starting in 2011 with AMD's ‘Bulldozer’ (Hollingsworth, 2012) architecture, and in 2013 with Intel's ‘Haswell’ (Intel, 2018) architecture, which provide fully IEEE-754-2008 compliant floating-point implementations. Even so, there is still no guarantee that a given compiler will make use of FMA operations. As FMA incurs a single rounding error but a separate multiply and add operation incurs two separate errors, results can depend on the compiler vendor and version. Transcendental functions are often implemented in software libraries, such as the glibc library used by GCC on Linux. Given that the error bounds for many of the glibc math functions have not been entered into the glibc manual (Loosemore, 2020) at the time of writing, it is difficult to determine whether results computed by these functions exactly match those computed by alternate math libraries. It is well-known that results depend on the library, and can even change in different versions of the same library. Tests have been done on values that are difficult to round correctly (Lefèvre, 2021) and (Zimmermann, 2021). Accurate libraries will compute matching results in general, but may return different results when the exact result is very close to the midpoint of consecutive floating-point numbers.

Another layer of complexity is added with the growing use of concurrency, for instance the massively parallel GPU accelerators popular in machine learning. The basic arithmetic functions of NVIDIA's compute unified device architecture (CUDA) (NVIDIA, 2018), including fused multiply-add, are fully IEEE-754-2008 compliant and even though the CUDA implementations of the transcendental functions are not necessarily correctly rounded, their error bounds are known and documented in the CUDA programming guide appendices (NVIDIA, 2018). However, CUDA also provides ‘intrinsic’ functions, which correspond to constructs of high-speed arithmetic circuits inside each CUDA core called Special Function Units (SFU). These intrinsic functions sacrifice accuracy in return for faster operation, affecting numerical error and hence reproducibility. In addition, the approach of massively parallel execution in itself poses new challenges in terms of reproducibility. Unlike in serial architectures there is often no guarantee on the order in which a pool of threads will finish working. Floating-point instructions may be ordered differently in separate runs of the same binary program and, since floating-point arithmetic lacks the associative property of real arithmetic, this can influence rounding error propagation. In combination with the known effects of catastrophic cancellations and other forms of error amplification this can lead to situations where results appear completely disparate in repeated runs of the same program on the same hardware. In such a situation, the normal paradigms of testing program validity against a reference implementation break down completely. It is worth noting that while this will be surprising to some, it is just making the known problem of otherwise hidden, potentially large numerical errors more visible.

#### 1.1.3. Error Bounding

In order to establish whether differences in numerical results are due to normal numerical errors as described above or due to algorithmic or programming errors, it would be useful to compute tight upper and lower bounds for the computed values with their expected numerical error. To determine error bounds, ‘range analysis’ methods are used, most prominently interval arithmetic (IA) and affine arithmetic (AA).

In interval arithmetic (IA), each of the floating-point variables are replaced with an interval variable $\bar{x}=\left[x_{a},x_{b}\right]$, each containing a lower and upper bound for its corresponding floating-point value. An alternative but equivalent representation is the centre-radius form $\bar{x}=\left[\left(x_{c}-x_{r}\right),\left(x_{c}+x_{r}\right)\right]$. The bounds of each interval variable are initialised with the same floating-point input value, and then track numerical errors so that the exact value of a given variable is guaranteed to be somewhere within its representative interval (Stolfi and de Figueiredo, 2007). Though fast and correct, IA suffers from the ‘dependency problem’ (Krämer, 2006), where IA treats variables as if completely unrelated even if they are dependent on each other. This can lead to unnecessarily loose error bounds. For instance, if $\bar{x}=\left[1,2\right]$, then $\bar{x}-\bar{x}=\left[\left(1-2\right),\left(2-1\right)\right]=\left[-1,1\right]$ even though the result should clearly be [0, 0]. Arithmetic expressions may be rewritten such that a variable only appears once on the right hand side, but this is usually not possible for complicated expressions.

Affine Arithmetic (AA) (de Figueiredo and Stolfi, 2004; Stolfi and de Figueiredo, 2007) is a range analysis method which aims to solve the dependency problem of IA by encoding correlations between variables within its range representation. Each affine range is a first-order polynomial. The constant term ${\hat{x}}_{c}$ represents the centre value of the range, in a manner similar to the IA centre-radius representation, but with *n* linear terms ${\hat{x}}_{\left[i\right]}\epsilon_{\left[i\right]}$ with *i* ∈ 1…*n* (henceforth deviation terms) instead of one.

(1) $$\begin{array}{l} \hat{x}={\hat{x}}_{c}+{\hat{x}}_{\left[1\right]}\epsilon_{\left[1\right]}+\ldots +{\hat{x}}_{\left[n\right]}\epsilon_{\left[n\right]},\;\epsilon_{\left[i\right]}\in \left[-1,1\right] \end{array}$$

These *n* deviation terms each represent a linear correlation with another variable. The values of all noise symbols ϵ_[*i*]_ are unknown, and they collectively represent the uncertainty in the interval. If they were known, then the exact error-free solution of a computation can be determined by simply substituting them into the above formula. Affine ranges $\hat{x}$ and ŷ are at least partially correlated if $\exists i>0:{\hat{x}}_{\left[i\right]}\neq 0\wedge {ŷ}_{\left[i\right]}\neq 0$. In other words, two affine ranges are correlated if they have non-zero deviation symbols in common. This means that the dependency problem and wrapping effect of standard IA is no longer an issue. For example, returning to the IA subtraction example again, where $\hat{x}=1.5+0.5\epsilon_{\left[1\right]}$ equivalently, we now have $\hat{x}-\hat{x}=\left(1.5+0.5\epsilon_{\left[1\right]}\right)-\left(1.5+0.5\epsilon_{\left[1\right]}\right)=0$ as desired. Since $\hat{x}$ is the same variable, and entirely correlated with itself, both occurrences of $\hat{x}$ share the deviation term ${\hat{x}}_{\left[1\right]}\epsilon_{\left[1\right]}$, which is allowed to cancel in the subtraction. This can happen with any affine forms which share the same deviation terms. Any numerical error from a function is simply appended to the resulting range as a new deviation term ${\hat{x}}_{\left[k\right]}\epsilon_{\left[k\right]}$, where ϵ_[*k*]_ is an unused noise symbol. The radius of an affine range is the sum of all absolute deviation coefficients in the interval ${\hat{x}}_{r}=\overset{n}{\underset{i=1}{\sum }}|{\hat{x}}_{\left[i\right]}|$. Since affine ranges are all first-order polynomials, AA can be considered a first-order range analysis method, where the error bounds are linear in ϵ. Any linear function of one or more affine ranges may be expressed exactly as another affine range by combining the midpoint and any corresponding error terms.

However, all nonlinear functions must be approximated to first order to be representable in AA - see Stolfi and de Figueiredo (2007). As a consequence, it is sometimes the case that AA multiplication, and consequently division, produce ranges that are wider than those computed with plain IA. This is a known weakness of the AA method, and is most likely to occur when operands are weakly or not correlated with each other, since AA only has the advantage when operands have shared noise symbols to cancel out. Alternative error estimates that give tighter ranges for affine multiplication are known. Based on the ideas presented in Bouissou et al. (2012), an improved affine multiplication error estimate is given in Equation 26 of Rump and Kashiwagi (2015), which we have implemented in Arpra.

Univariate non-linear functions can be approximated by two common methods; the Chebyshev and Min-Range approximations. In brief, the goal is to determine values that lead to the best linear approximation of a function *f* on the input range $\left[x_{a},x_{b}\right]=\left[\left({\hat{x}}_{c}-{\hat{x}}_{r}\right),\left({\hat{x}}_{c}+{\hat{x}}_{r}\right)\right]$. These values, which depend explicitly on both the function *f* and the input range $\hat{x}$, are α, γ and δ. With these values, the resulting range ŷ can be computed as $f\left(\hat{x}\right)=\alpha \hat{x}+\gamma +\epsilon_{\left[k\right]}\delta =\left(\alpha {\hat{x}}_{c}+\gamma \right)+\epsilon_{\left[1\right]}\left(\alpha {\hat{x}}_{\left[1\right]}\right)+\ldots +\epsilon_{\left[n\right]}\left(\alpha {\hat{x}}_{\left[n\right]}\right)+\epsilon_{\left[k\right]}\delta$, where ϵ_[*k*]_ is a new unused noise symbol. However, the meaning of the best approximation is ambiguous, as one can either minimise the error δ or minimise the overall range of the result. The so-called Chebyshev approximation minimises the error and the Min-Range approximation the resulting range. See Stolfi and de Figueiredo (2007) for details on how these values are computed for each approximation method.

The Chebyshev approximation is the theoretically ideal option, since AA is best when as much correlation information as possible can be preserved, and as little approximation error as possible introduced. However, the Chebyshev approximation suffers from the ‘overshoot’ and ‘undershoot’ phenomenon, where the range of the computed result is bigger than if it were computed in IA (Stolfi and de Figueiredo, 2007). This is especially problematic when approximating over larger input ranges. To prevent overshoot and undershoot, the Min-Range approximation can be used. At the expense of some correlation information, thus a larger independent error term δ, one can find a function which approximates the non-linear function as tightly as plain IA does.

## 2. Materials and Methods

We here present Arpra, which is an open source library for **Ar**bitrary-**p**recision **r**ange **a**nalysis, written in C. Its compatible with all UNIX-like operating systems, including Linux, BSD and macOS, and is licensed under the terms of the GNU Lesser General Public version 3 license (LGPL-3.0). Arpra is primarily intended to be a diagnostic tool for debugging behavioural changes in numerical simulations caused by the introduction and propagation of numerical error. It implements a modified version of the mixed IA/AA range analysis method, described in Stolfi and de Figueiredo (2007) and Rump and Kashiwagi (2015), implemented in INTLAB (Rump, 1999). In addition, it has novel space and time saving truncation procedures as discussed below. Arpra uses GNU MPFR (Fousse et al., 2007) as its floating-point back end. MPFR has many advantages over standard floating-point implementations, such as the ability to set any variable's precision dynamically, and to choose the rounding mode on a per-operation basis without a costly FPU register setting operation.

### 2.1. Features of the Arpra Library

The Arpra library loosely follows the design philosophy of the MPFR library (Fousse et al., 2007) and represents ranges with C structures. The elementary structure of an Arpra computation is known as an arpra_range.


  struct arpra_range_struct {
      mpfr_prec_t    precision;
      __mpfr_struct  centre;
      __mpfr_struct  radius;
      __mpfi_struct  true_range;
      unsigned int  *symbols;
      __mpfr_struct *deviations;
      unsigned int   nTerms;
  };
  
  typedef struct arpra_range_struct
  arpra_range;


The precision field stores the range's ‘working precision’. The centre and radius fields, respectively, hold the centre and radius values of the range. The true_range field is an MPFI interval representing the actual lower and upper bounds of the range, in the working precision. MPFI is an implementation of IA, written by Revol and Rouillier (2005), which also uses MPFR as its floating-point back end. Next, the symbols and deviations fields are, respectively, pointers to an array of noise symbol numbers and a corresponding array of deviation coefficients, and nTerms is the number of deviation terms currently stored in the symbols and deviations arrays. The radius field is a redundant variable which accumulates the absolute value of all deviation terms in the arpra_range. The radius must be known internally by Arpra in a few places, including when computing the true_range field, but this field could in principle be computed on demand, saving the space of one MPFR number per arpra_range instance in memory. Throughout the remainder of this document, for any arpra_range variable $\hat{x}$, centre, radius and true_range are, respectively, denoted $\hat{x}.c$, $\hat{x}.r$ and $\hat{x}.t$, while deviations, symbols and nTerms are denoted $\hat{x}.d$, $\hat{x}.s$ and $\hat{x}.n$.

In an AA implementation which accounts for rounding errors, a new deviation term is typically added after each operation leading to rapid growth in the number of active noise symbols, but each noise symbol often only affects a small subset of active affine ranges. In an effort to reduce the memory footprint of AA, we use a sparse representation of non-zero deviation coefficients inside the deviations array, following Stolfi and de Figueiredo (2007). For each deviation coefficient, the corresponding noise symbol number is stored at the same index of the symbols array, as the following example shows.

(2) $$\begin{array}{l} \begin{array}{c} \text{deviations}=\left(2.45,1.03,12.56,3.12\right)\\ \text{ symbols}=\left(1,3,4,6\right) \end{array} \end{array}$$

The deviation terms stored in these arrays are sorted in order of increasing noise symbol number, to reduce the complexity of indexing into them. In the above example, note how at least six noise symbol numbers exist globally: (1, 2, 3, 4, 5, 6). However, only the four symbols (1, 3, 4, 6) are actually stored in the deviation term arrays, since the deviation coefficients of symbol numbers (2, 5) are zero. Depending on the number of active noise symbols at a given point in the computation, this could be far less computationally intensive than the equivalent dense representation.

Each arpra_range must be initialised before use using either arpra_init or arpra_init2. The former initialises an arpra_range with default working precision, while the latter initialises it with a given working precision. This allocates the internal memory of the range, and initialises it to the Arpra equivalent of IEEE-754 not-a-number (henceforth NaN). When done with a range, the memory should be freed to prevent memory leaks using arpra_clear.

#### 2.1.1. Function Structure

Arpra implements the plus, minus, negation, multiplication and division operations, as well as the Chebyshev versions of the square root, natural exponential, natural logarithm and multiplicative inverse (reciprocal) functions as implemented in de Figueiredo and Stolfi (2004). Arpra also implements Min-Range versions of the natural exponential and multiplicative inverse functions, with Min-Range square root and natural logarithm left for future work. Arpra mathematical functions use a function schema similar to that used by MPFR, with the result pointer followed by the operand pointers. For instance, bivariate function calls look like arpra_f2(y_ptr, const x1_ptr, const x2_ptr). All Arpra functions first check for domain violations. For instance, if computing the square root of a range that contains negative numbers, then a NaN range is returned. Like most (but not all) functions in regular floating-point arithmetic, Arpra functions produce NaN ranges if any of the operand ranges are NaN. A range is considered NaN if any of the true_range bounds are NaN. A range is infinity if the true_range bounds are ±∞, and neither of them are NaN. If either of the operands are NaN or infinity, then the function immediately sets the result to, respectively, NaN or infinity, and then returns, skipping many unnecessary instructions. Next, the new centre value ŷ.*c* and the deviation coefficients ŷ.*d* are computed, along with the new numerical error term. The absolute values of these coefficients are accumulated in the radius, rounding upwards, using the mpfr_sum function. For the nonlinear univariate functions, the floating-point approximation parameters α and γ are computed using the true_range field of the input $\hat{x}$ as MPFI intervals (Revol and Rouillier, 2005). The resulting range's centre ŷ.*c* and deviation coefficients ŷ.*d* are then computed using the centre values of these MPFI intervals, and the radii of α and γ are added to the numerical error term δ, rounding upwards. Next, the new true_range field is computed, and excess error is optionally removed by the mix_trim procedure, as discussed below.

#### 2.1.2. Arbitrary-Precision

Like MPFR itself, for its variables, Arpra allows users to dynamically change the precision field of arpra_range variables (used to set the precision of the true_range field), which is useful for determining the effect of altering floating-point precision in a computation. Arbitrary precision is also useful for calculating intermediate quantities more precisely. When computing a new arpra_range, any overhead rounding error (incurred from internal Arpra floating-point operations) must be accumulated in the new numerical error deviation term, increasing the range's width. Therefore, we want these computations to be as accurate as possible, such as to minimise this overhead rounding error. The Arpra library achieves this by computing and storing ŷ.*c* and ŷ.*d* in an extended global ‘internal precision’, which is higher than the working precision of all ranges currently in use. Intermediate quantities, including the α and γ approximation parameter intervals, are also computed in this internal precision. We can do this safely because only the true_range field is required to be rounded to the specified working precision; all computations up until the final rounding can be done in whichever precision one chooses, so it makes sense to choose a higher one.

The working precision of an arpra_range (the precision of its true_range field) is set during initialisation. If a range is initialised using the arpra_init2 function, then its working precision is set to the value of the precision argument. If the range is initialised with the arpra_init function, then its working precision is determined by a global ‘default precision’ variable. The default precision can be retrieved using the arpra_get_default_precision function, and dynamically set by the user using the arpra_set_default_precision function. One can also retrieve and dynamically set the working precision of a range that has already been initialised by using arpra_get_precision and arpra_set_precision. Setting the precision of a range using the above setter function is faster than clearing and reinitialising it. Note, however, that setting it in this manner causes the range to be reset to NaN. If one needs to change the precision of a range without invalidating it, one can simply initialise a new range with the desired precision, and then set the new range with the old one using the arpra_set function. As with the default precision, and the precision of individual ranges, the user is able to retrieve and dynamically set this internal precision using the arpra_get_internal_precision and arpra_set_internal_precision functions.

#### 2.1.3. Mixed Trimmed AA/IA

We have discussed how arbitrary-precision can help us to minimise the overhead rounding error caused by the AA method. However, since AA ranges are essentially first-order polynomials, often with many deviation terms each, a small amount of overhead rounding error is inevitable when computing the true_range. Furthermore, approximation error from multiplication and the transcendental functions can result in ranges that are wider than those computed with plain IA, regardless of rounding error. To reduce the impact of these additional error sources, Arpra implements a modified version of the mixed IA/AA method. In plain AA, the true_range field of a range ŷ is simply the interval ŷ.*t* = [(ŷ.*c* − ŷ.*r*), (ŷ.*c* + ŷ.*r*)], rounded outwards in working precision. As a consequence, the rounding error from these bound calculations, and the error incurred in nonlinear function approximation, is included as bloat in the final true_range. In order to trim some of this excess error, a method known as ‘mixed IA/AA’ can be used. This method is described by Stolfi and de Figueiredo (2007), and Rump and Kashiwagi (2015), and has been implemented in INTLAB (Rump, 1999).

The idea of mixed IA/AA is to simultaneously compute AA and IA versions of each range, and use the information from the IA method to complement the AA method. Arpra uses the MPFI library (Revol and Rouillier, 2005) for IA functions. Specifically, when computing some AA function on affine ranges, the IA version of that function is also computed on the true_range fields of those affine ranges. After that, the true_range field of the resulting range is taken to be the intersection of the AA range ŷ.*t* and the IA range ȳ. Doing this consistently ensures that the true_range field of all resulting ranges is never worse than when computing it with either AA or IA on their own. In other words, if variable correlations cause an IA range to expand, the AA range will compensate. Conversely, if approximation error causes an AA range to expand, the IA range compensates. We emphasise that only the true_range is modified in mixed IA/AA, while the centre and deviation terms remain the same.

However, one can do better than this. The Arpra library implements a modified version of mixed IA/AA, which we will refer to as ‘mixed trimmed AA/IA’. In this version, if the interval *T* = [(ŷ.*c* − ŷ.*r*), (ŷ.*c* + ŷ.*r*)] fully contains the interval ŷ.*t* computed with the mixed IA/AA method, then the new error deviation term of the AA operation can also be trimmed a little, so long as we maintain the condition that *T* ⊇ ŷ.*t*, and that the new error term is reduced to a minimum of zero. It is safe to do this because the new error term is an independent deviation term, which noise symbol number is not used elsewhere in the computation, so no correlation information is lost. The complete Arpra range mixing and trimming procedure is shown in Algorithm 1, where RD and RU means round down and up, respectively. This procedure assumes that the approximated function is twice differentiable and that its second derivative does not change sign within the approximation interval (i.e., the function is convex or concave within the approximation interval). See the Appendix for proofs that range correctness is maintained by mix_trim for Chebyshev and Min-Range approximations of functions that fulfill these criteria.

**Algorithm 1:**
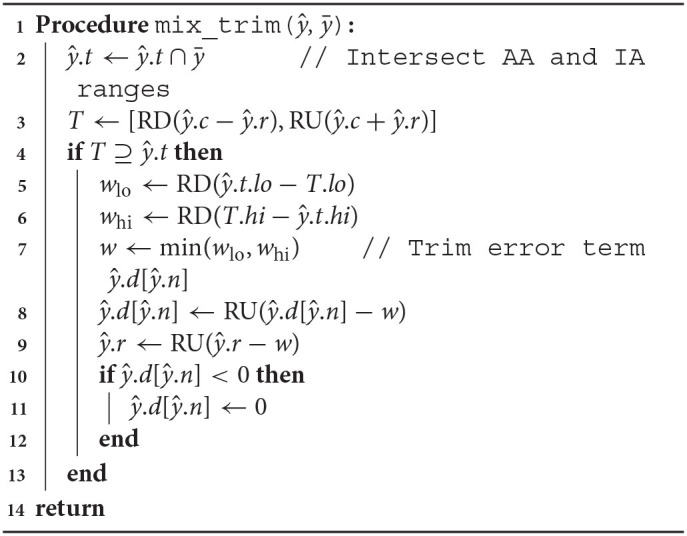
Algorithm for combining AA and IA ranges and trimming the error term.

#### 2.1.4. Term Reduction Functions

In long computations, it is often the case that the number of deviation terms accumulated in AA ranges becomes very large, and an AA computation can often grind to a halt after a short while, due to the computational overhead. No other AA implementation handles this eventuality, to our best knowledge, even though a known solution exists (Stolfi and de Figueiredo, 2007). A so-called ‘term condensing’ function, which sums the absolute value of selected deviation coefficients into a new coefficient, corresponding to a new noise symbol ϵ_[*k*]_, and removes the replaced terms. For example, if one has an AA range $\hat{x}$, with deviation terms (1.5ϵ_[1]_, 8ϵ_[2]_, 2ϵ_[3]_, −4ϵ_[4]_, 1ϵ_[5]_), one can reduce the ϵ_[1]_, ϵ_[3]_ and ϵ_[4]_ terms of $\hat{x}$ in a new range ŷ, with just three deviation terms.

(3) $$\begin{array}{l} ŷ={\hat{x}}_{c}+8\epsilon_{\left[2\right]}+1\epsilon_{\left[5\right]}+\left(\left|1.5|+|2|+|-4\right|\right)\epsilon_{\left[k\right]} \end{array}$$

Although some of the correlation information in $\hat{x}$ is potentially lost in ŷ, this is a safe operation, since ϵ_[*k*]_ is a new and independent noise symbol, and the actual range ŷ_*c*_ ± ŷ_*r*_ of ŷ is not smaller than the range of $\hat{x}$.

Arpra provides three variants of a term condense function. arpra_reduce_last_n condenses the last *n* deviation terms of a range, while arpra_reduce_small_abs function reduces all terms which deviation magnitude is less than or equal to a given threshold and arpra_reduce_small_rel reduces all terms whose deviation magnitude is less than or equal to a given fraction of the range's radius. The arpra_reduce_last_n function, listed in Algorithm 2, can be considered a ‘lossless' condensing function, if used correctly, that is to say that, if the noise symbols in the last *n* deviation terms are not present in any other range, this function is guaranteed to preserve all correlation information when condensing terms. There are a number of situations in which the last *n* terms of a range are independent. For instance, if only a single arpra_range is returned by any given function, then all noise symbols introduced by the intermediate computations in that function are guaranteed to be only present in the returned range. Alternatively, one can use arpra_reduce_small_abs, listed in Algorithm 3, or arpra_reduce_small_rel, listed in Algorithm 4, if some loss of correlation information is acceptable. These condensing functions can be considered ‘lossy', since there is no direct control over which deviations terms are condensed, and some of these terms may consequently be correlated ones. However, this matters less when the deviation coefficients are small. If the majority of deviation coefficients are close to zero, with just a few coefficients contributing to the majority of the radius, then the loss of correlation information will be minimal when these low magnitude terms are condensed.

**Algorithm 2:**
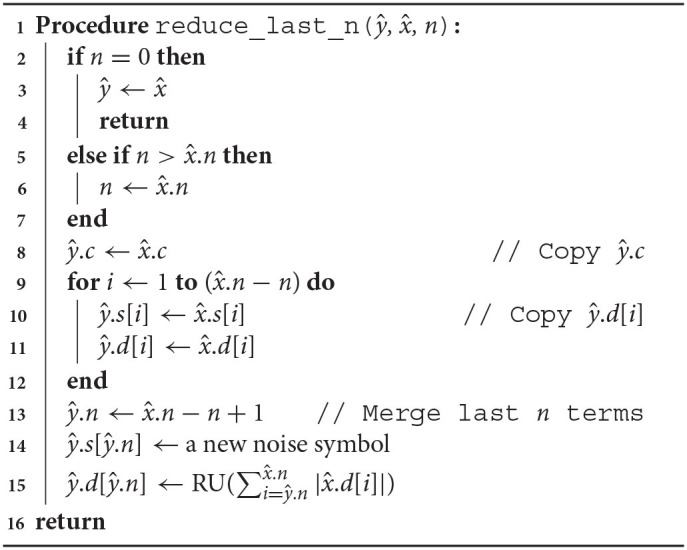
Condense the last *n* terms

**Algorithm 3:**
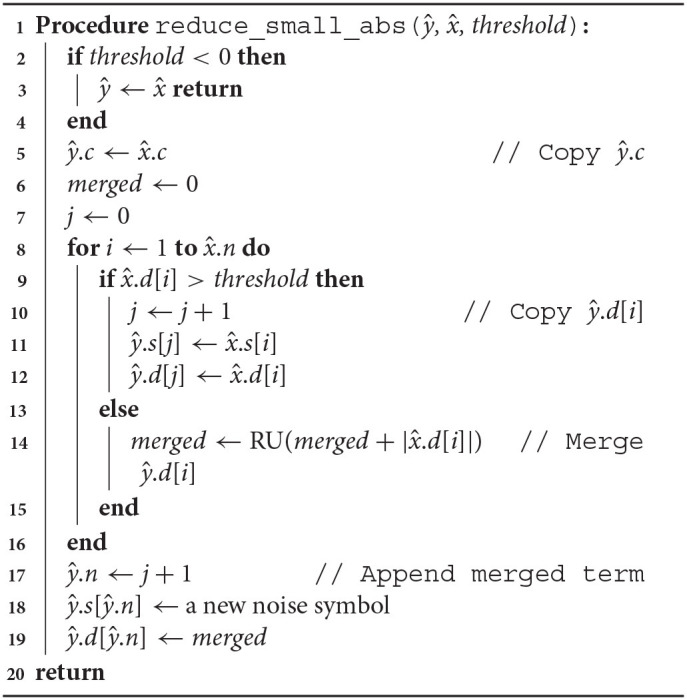
Condense terms smaller than *threshold*

**Algorithm 4:**
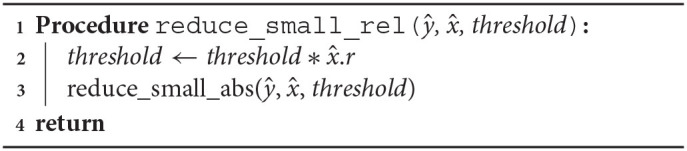
Condense terms smaller than $\hat{x}$.*r* · *threshold*

### 2.2. Neural Network Model

We test Arpra on prototypical neural network models as described in the results below. Within the network we use either the Morris-Lecar neuron model (Morris and Lecar, 1981) or the Traub-Miles (Hodgkin-Huxley) model (Traub and Miles, 1991).

The Morris-Lecar model is defined as follows:

(4) $$\begin{array}{l} C\frac{dV}{dt}=I-g_{\text{Ca}}m_{\infty }\left(V\right)\left(V-V_{\text{Ca}}\right)\\ \;-g_{\text{K}}n\left(V-V_{\text{K}}\right)-g_{\text{L}}\left(V-V_{\text{L}}\right)\\ \;\frac{dn}{dt}=\frac{n_{\infty }\left(V\right)-n}{\tau_{n}\left(V\right)}, \end{array}$$

using the following auxiliary functions:

(5) $$\begin{array}{l} m_{\infty }\left(V\right)=\frac{1+\tanh\left(\frac{V-V_{1}}{V_{2}}\right)}{2}\\ n_{\infty }\left(V\right)=\frac{1+\tanh\left(\frac{V-V_{3}}{V_{4}}\right)}{2}\\ \tau_{n}\left(V\right)=\frac{1}{\phi \cosh\left(\frac{V-V_{3}}{2V_{4}}\right)}. \end{array}$$

where *V* the membrane potential and *n* the probability of rectifying K^+^ ion channels opening. *m*_∞_ and *n*_∞_ are the steady state value for *m* and *n*, respectively, where *m* is the probability of depolarising Ca^2+^ ion channels opening. *g*_Ca_, *g*_K_ and *g*_L_ are conductance values for calcium, potassium and leak channels, respectively, while *V*_Ca_, *V*_K_ and *V*_L_ are their respective reversal potentials. *I* represents current inputs from external sources, *C* is the cell membrane capacitance, ϕ is the rate of the recovery process, and the *V*_1_, …, *V*_4_ parameters determine the shape of the steady state activation curves for *m* and *n*, and the *n* time scale. The parameters were set such that the neurons exhibit class 1 excitability Morris and Lecar (1981), in particular *g*_L_ = 2 μS, *g*_Ca_ = 4 μS and *g*_K_ = 8 μS, *V*_L_ = −60 mV, *V*_Ca_ = 120 mV, and *V*_K_ = −80 mV. The remaining parameters are *V*_1_ = −1.2, *V*_2_ = 18, *V*_3_ = 12, *V*_4_ = 17.4, ϕ = 1/15 and *C* = 20 nF.

The Traub-Miles model (Traub and Miles, 1991) is defined as:

(6) $$\begin{array}{l} \begin{array}{c} \;C\frac{dV}{dt}=I-I_{\text{L}}-I_{\text{Na}}-I_{\text{K}}\\ \;I_{\text{L}}=g_{\text{L}}\left(V-V_{\text{L}}\right)\\ \;I_{\text{Na}}=g_{\text{Na}}m^{3}h\left(V-V_{\text{Na}}\right)\\ \;I_{\text{K}}=g_{\text{K}}n^{4}\left(V-V_{\text{K}}\right)\\ \frac{dy\left(t\right)}{dt}=\alpha_{Y}\left(V\right)\left(1-Y\right)-\beta_{Y}\left(V\right)Y, \end{array} \end{array}$$

where *Y* ∈ {*m, h, n*}, and

(7) $$\begin{array}{l} \begin{array}{c} \alpha_{m}\left(V\right)=0.32\left(-52-V\right)/\left(\exp\left(\left(-52-V\right)/4\right)-1\right)\\ \beta_{m}\left(V\right)=0.28\left(25+V\right)/\left(\exp\left(\left(25+V\right)/5\right)-1\right)\\ \alpha_{h}\left(V\right)=0.128\exp\left(\left(-48-V\right)/18\right)\\ \beta_{h}\left(V\right)=4/\left(\exp\left(\left(-25-V\right)/5\right)+1\right)\\ \alpha_{n}\left(V\right)=0.032\left(-50-V\right)/\left(\exp\left(\left(-50-V\right)/5\right)-1\right)\\ \beta_{n}\left(V\right)=0.5\exp\left(\left(-55-V\right)/40\right). \end{array} \end{array}$$

*m* is the probability of Na^+^ channel activation, *h* is the probability that Na^+^ channels are not blocked, and *n* is the probability of K^+^ channel activation The chosen parameters are typical for this model, with *g*_L_ = 0.02672 μS, *V*_L_ = −63.563 mV, *g*_Na_ = 7.15 μS, *V*_Na_ = 50 mV, *g*_K_ = 1.43 μS, *V*_K_ = −95 mV and *C* = 0.143 nF.

The synapses of the SNN model are simulated using a model similar to the standard Rall synapse (Rall, 1967), but with the additional constraint of being fully continuous.

(8) $$\begin{array}{l} \begin{array}{c} \frac{dR}{dt}=\alpha \sigma \left(V_{\text{pre}}-V_{\text{thr}}\right)-\beta R\\ \frac{dS}{dt}=\gamma R-\delta S, \end{array} \end{array}$$

where σ is the sigmoid function

(9) $$\begin{array}{l} \sigma \left(x\right)=\frac{1}{1+e^{-kx}} \end{array}$$

and *k* is the steepness of the synapse activation slope. In the results, for simplicity, α = γ and β = δ. The receptor activation *S* causes a postsynaptic current *I*_syn_ according to

(10) $$\begin{array}{l} I_{\text{syn}}=g_{\text{syn}}S\left(V_{\text{syn}}-V\right) \end{array}$$

where *g*_syn_ is the synaptic conductance and *V*_syn_ is the reversal potential. For the Morris-Lecar model experiments, the conductance of each synapse was drawn from a normal distribution with standard deviation 1 and mean 150/*n*_pre_, where *n*_pre_ is the number of presynaptic neurons. For the Traub-Miles model experiments, the conductances are drawn from a normal distribution with both standard deviation and mean equal to 1.3/*n*_pre_. The remaining parameters are set to *V*_syn_ = 0 mV, *V*_thr_ = −50 mV, α = 0.25 kHz, β = 0.15 kHz and *k* = 10^6^.

In order to create biologically plausible SNN models, randomised input spikes are generated by dummy Poisson neuron models, which are then propagated to the Morris-Lecar neuron models via the modified Rall synapses. Each Poisson neuron is modelled as a Poisson point process.

(11) $$\begin{array}{l} P\left(N\left(\Delta t\right)=n\right)=\frac{{\left(0.001\lambda \Delta t\right)}^{n}}{n!}e^{-0.001\lambda \Delta t} \end{array}$$

where *P*(*N*(Δ*t*) = *n*) is the probability of *n* spike events occurring within the next timestep, and λ is the desired spike rate in Hz. For the small timesteps used here, the probability of more than one spike per timestep can be neglected and we use the approximation

(12) $$\begin{array}{l} P\left(N\left(\Delta t\right)=0\right)=e^{-0.001\lambda \Delta t}P\left(N\left(\Delta t\right)=1\right)=1-P\left(N\left(\Delta t\right)=0\right) \end{array}$$

We sample distribution for each neuron and if it spikes, *V* is set high to 20 mV, otherwise it is set low to −60 mV.

## 3. Results

### 3.1. Accuracy of the Arpra Library

In order to test the accuracy of ranges computed by Arpra, compared to those computed with IA, we computed result ranges ŷ with Arpra arithmetic functions *n* = 100, 000 times on randomly generated operands ${\hat{x}}_{1}$ and ${\hat{x}}_{2}$, each with centre values drawn from a uniform distribution in [100, 500], and between zero and nine deviation terms drawn from a uniform distribution in [−10, 10]. The IA result ȳ was computed on the true_range fields ${\hat{x}}_{1}.t$ and ${\hat{x}}_{2}.t$ of these operands using the corresponding arithmetic function from the MPFI library (Revol and Rouillier, 2005). For each test, the diameter of the Arpra result *D*_Arpra_ = ŷ.*t*.*hi*−ŷ.*t*.*lo* relative to the diameter of the IA result *D*_IA_ = ȳ.*hi*−ȳ.*lo* is computed as *D*_rel_ = *D*_Arpra_/*D*_IA_. Tests were performed both for plain AA and for mixed IA/AA. The working precision of all test cases was 24, corresponding to IEEE-754 single-precision numbers, while Arpra's internal precision was set to 256. All transcendental functions used the Chebyshev approximation method.

**Figure 1:**
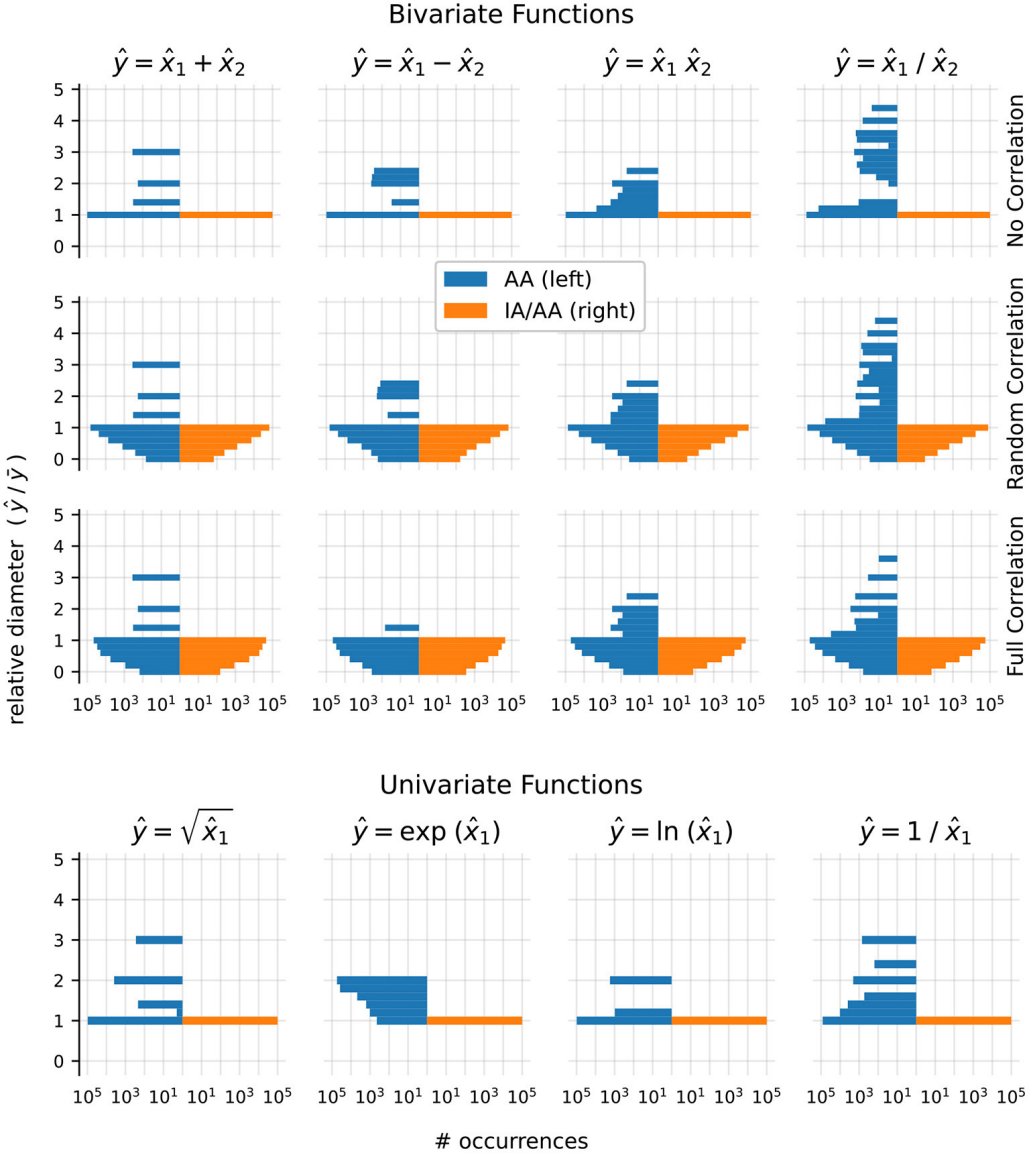
Plots of Arpra diameters relative to IA diameters. The left half of each plot shows results using the plain AA method, while the right side shows the mixed IA/AA method. The top three rows show results for the bivariate functions addition, subtraction, multiplication and division, each with no correlation, random correlation and full correlation of operands. The bottom row shows results for the univariate functions square root, exponential, logarithm and inverse function. Please note the logarithmic scale on the x-axis.

Univariate functions were tested once for each test case. Bivariate functions were tested three times for each test case, with different operand correlation scenarios, in order to determine how the strength of operand correlation affects the resulting range. In the no correlation scenario, the noise symbol sets ${\hat{x}}_{1}.s$ and ${\hat{x}}_{2}.s$ of the operands are mutually exclusive. In the random correlation scenario, each pair of noise symbols $\left({\hat{x}}_{1}.s\left[i\right],{\hat{x}}_{2}.s\left[i\right]\right)$, with $i\leq \min\left({\hat{x}}_{1}.n,{\hat{x}}_{2}.n\right)$, contained identical symbols with probability 0.5. In the full correlation scenario, all noise symbol pairs $\left({\hat{x}}_{1}.s\left[i\right],{\hat{x}}_{2}.s\left[i\right]\right)$ contained identical symbols. The relative diameters of Arpra ranges are shown in Figure 1.

The observed relative diameters of bivariate functions clearly illustrate how deviation term cancellation improves the resulting range when operands are correlated. Note how the distribution of relative diameters progressively moves towards zero as operand correlation increases. With the plain AA method, although the majority of linear addition and subtraction result ranges are at least as good as IA ranges, they can sometimes be wider. This is more noticeable as the ratio of overhead rounding error to range diameter increases. However, deviation term cancellation leads to large improvements over IA results when operands are correlated. The same applies to the nonlinear multiplication and division functions, although the extra linearisation error increases the chance of range overestimation. However, with the mixed IA/AA method, the relative diameter of all results is bounded to a maximum of one, due to the range intersection step in Algorithm 1.

Ranges computed by nonlinear univariate functions are also subject to linearisation error. However, with no opportunity for deviation term cancellation, the relative diameter of these ranges is at least one with the plain AA method. The plain AA exponential function results in Figure 1 show especially large relative diameters, since the Chebyshev exponential approximation is prone to undershoot in this input domain. With the mixed IA/AA method, however, all resulting ranges are exactly equal to their IA counterparts.

### 3.2. The Hénon Map

In this section, we next tested the performance of the Arpra library on a simulation of the Hénon map (Hénon, 1976), which is a dynamical system with known stability properties in different dynamical regimes. The Hénon map has trajectories ranging from stable limit cycles to chaotic attractors, depending on the choice of parameters. In addition to evaluating Arpra, this allows us to observe how system stability affects the growth of Arpra ranges. The model was used in Rump and Kashiwagi (2015) to test the INTLAB range analysis package for MATLAB, making it a good first benchmark to see how the Arpra library compares, given its additional arbitrary internal precision and term condensing functions.

The Hénon map is defined by the following equations, where *x*_*i*_ and *y*_*i*_ are the state variables at the *i*th iteration, while α and β are constant parameters.

(13) $$\begin{array}{l} \begin{array}{c} x_{i+1}=1-\alpha x_{i}^{2}+y_{i}\\ y_{i+1}=\beta x_{i} \end{array} \end{array}$$

In the ‘classical’ Hénon map, α = 1.4 and β = 0.3, resulting in a chaotic system. However, the system is also known to have a stable periodic orbit below around α = 1.06, and is increasingly stable as α is reduced further. Note that transcendental functions are not required to implement this model. As a consequence, the only sources of overhead error from the AA method are floating-point rounding errors and approximation errors from multiplication. For all experiments in this section, both *x* and *y* are initialised as ranges centred on zero, with small initial radii of 1*e* − 5, the β parameter is fixed to 0.3, and a working precision of 53 is used. All simulations use version 0.2 of the Arpra library (Turner, 2019).

**Figure 2:**
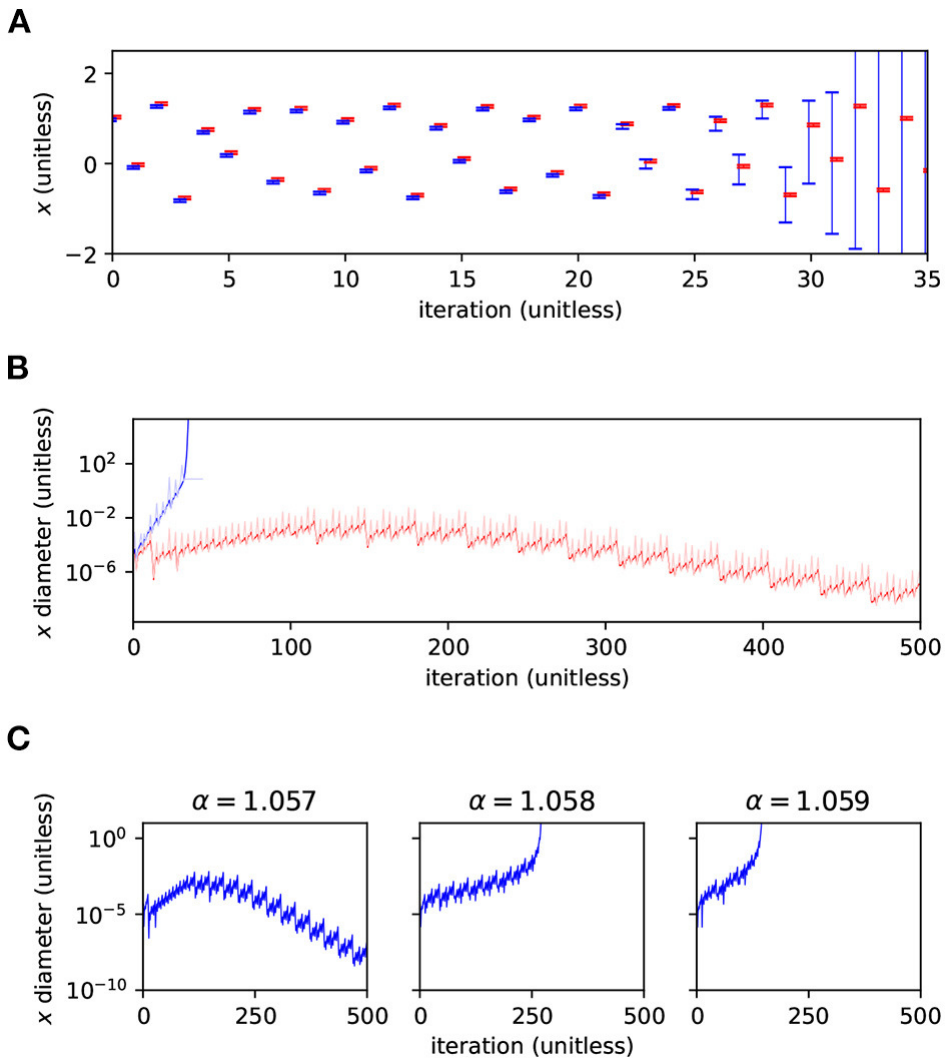
Range analysis of the Hénon map's *x* variable. **(A)** Values of *x* and the range (errorbars) computed by Arpra using plain AA (red), and by MPFI using IA (blue). Note that the symbols were offset diagonally to make them visible where they coincide. **(B)** Diameter of the range, in log scale, for 500 iterations (red, blue) and the diameter relative to the magnitude of the range's centre (light red, light blue). Note how the IA ranges begin to explode at around *i* = 30, while the AA ranges shrink after a short growth period. α = 1.057 and β = 0.3 in **(A,B)**. **(C)** Diameter (log scale) of the *x* range computed using Arpra's plain AA method for α = 1.057 (left), α = 1.058 (centre) and α = 1.059 (right).

We first compared the plain AA method of Arpra with the IA method of the MPFI library (Revol and Rouillier, 2005). The system was iterated for *n* = 500 steps with Arpra's internal precision set to 53, equal to the working precision. The α parameter of the Hénon map was set to 1.057, meaning the model was close to chaotic, but still locally stable. The *x* ranges of the AA and IA runs are shown in Figures 2A,B. The *y* ranges behave similarly (data not shown). The figure illustrates that the ranges computed in IA explode almost immediately to infinite width after only about *i* = 30 iterations, despite the global stability of the underlying model. In agreement with Rump and Kashiwagi (2015), the ranges computed with AA initially grow for a short while, but then begin to shrink back below their initial width from around iteration *i* = 100, as the trajectory converges to a periodic orbit.

Trajectories in chaotic dynamical systems are, by definition, highly sensitive to perturbations in the initial state, and these perturbations can propagate in unpredictable ways. As a result, ranges representing the state of these systems can grow very quickly. The Hénon map is known to exhibit chaotic behaviour with β = 0.3 and α approaching around 1.06. So, we next tested α = 1.057, 1.058 and 1.059, to see how changes in the local stability of the Hénon map affect the diameter of computed ranges. Arpra's plain AA method was used with internal precision *p* = 53. The range diameter for the Hénon map *x* variable is shown for each α value in Figure 2C. In the left column, as we saw earlier, the diameter of ranges computed in the stable Hénon map initially grows, as the trajectory converges to its stable orbit, but begins to shrink once the stable orbit is reached. As α is increased, the Hénon map enters a chaotic regime, and the small perturbations represented by the affine ranges are amplified in unpredictable ways. This results in the runaway growth of the bounding range (Figure 2C middle and right panel). The rate of range growth is dependent on how sensitive, or rather how chaotic, the system is. Higher values of α result in faster range growth.

This effect can pose a problem for analysing systems with singularities. For example, in range analysis methods such as IA and AA, dividing by a range which straddles zero results in an infinite width range, since values from the denominator range can be arbitrarily close to zero. If one analyses a sufficiently unstable system involving division, it is possible that the computed ranges will quickly grow large enough such that they eventually straddle zero, resulting in immediate range explosion. Even if ranges which straddle zero do not occur, there is still the question of whether ranges with such large diameters are useful in practice. Thus, the analysis of highly unstable systems with Arpra, or indeed any AA implementation, is not recommended. This issue is not unique to IA and AA. Statistical approaches to error bounding, such as discrete stochastic arithmetic (Vignes, 2004), would also produce garbage results in unstable computations. Using a higher working precision may help to mitigate the issue, but only temporarily.

**Figure 3:**
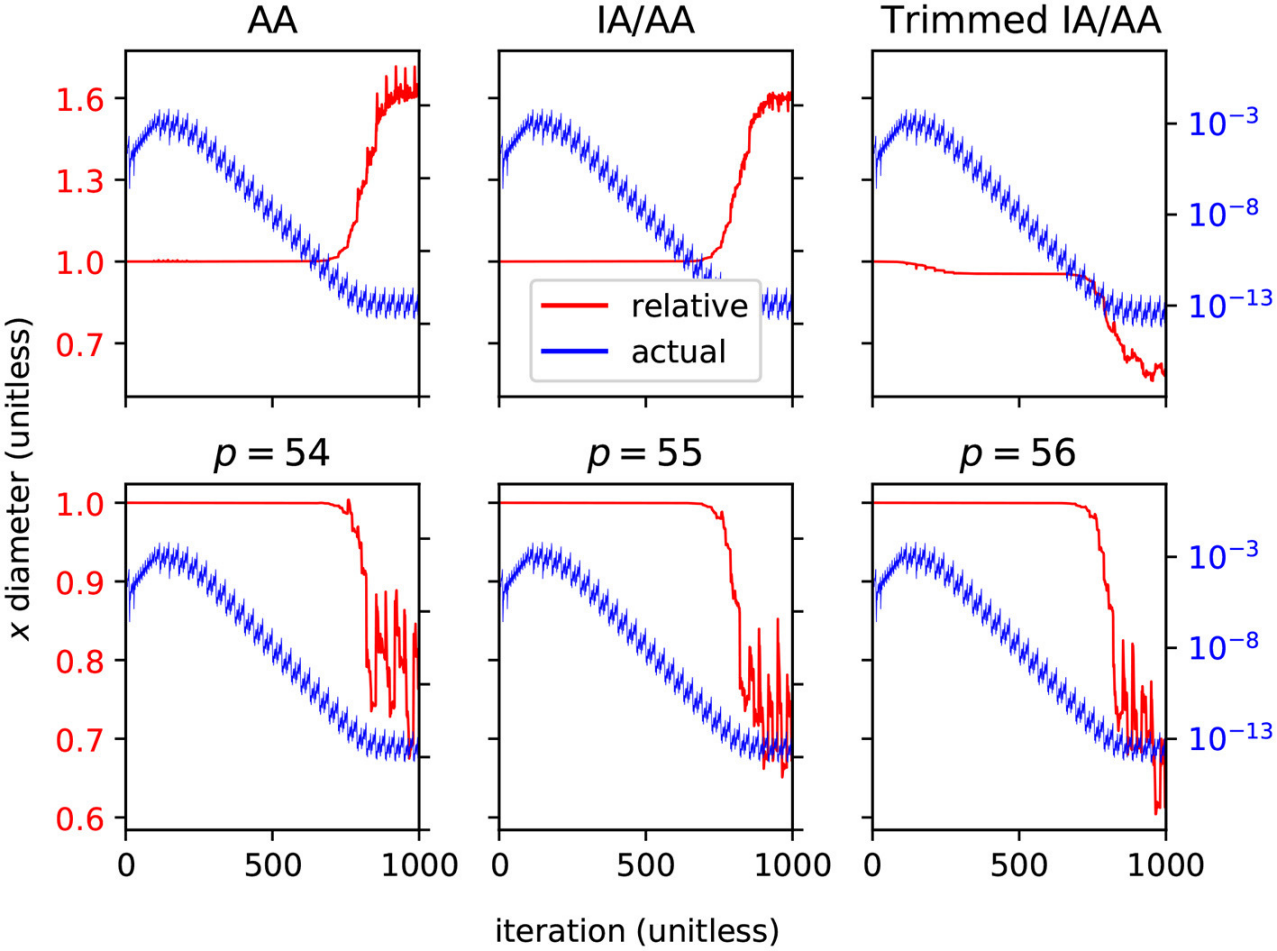
Relative diameters (red, linear scale) and actual diameters (blue, log scale) of Arpra ranges representing the Hénon map's *x* variable. The top plots show diameters of Arpra ranges computed with the AA, mixed IA/AA and mixed trimmed IA/AA methods, where relative diameter plots are relative to INTLAB mixed IA/AA range diameters. The bottom plots show diameters of Arpra mixed trimmed IA/AA ranges computed with internal precision set to *p* = 54, *p* = 55 and *p* = 56, where relative diameter plots are relative to Arpra range diameters with internal precision set to *p* = 53.

Arpra is capable of more advanced methods than just plain AA, with higher internal precision. It is expected that ranges computed with these advanced features should be comparatively tighter for a small runtime and memory cost. To investigate this, we iterated the Hénon map for *n* = 1, 000 steps, with α = 1.057. To evaluate Arpra's range analysis methods, we compare the diameter of Arpra ranges computed with AA, mixed IA/AA and mixed trimmed IA/AA to those of reference ranges computed in an equivalent simulation using INTLAB (Rump, 1999) version 11. Other affine arithmetic packages also exist, such as yalAA (Kiel, 2012) and kv (Kashiwagi, 2020). We chose to compare against INTLAB since it is widely known, and readily implements the mixed IA/AA method. To compare ranges fairly, Arpra's internal precision was set to 53, matching the double-precision numbers used internally by INTLAB. Arpra results are shown in the top row of Figure 3. To evaluate Arpra's extended internal precision feature, we compare Arpra mixed trimmed IA/AA range diameters computed with internal precision set to *p* = 54, *p* = 55 and *p* = 56 to diameters of reference Arpra mixed trimmed IA/AA ranges. The reference ranges are computed with internal precision *p* = 53, equal to the working precision. Internal precision results are shown in the bottom row of Figure 3. In all plots, Arpra range diameters are plotted in blue, while Arpra range diameter divided by reference range diameter is plotted in red.

As the top left and top middle plots of Figure 3 show, Arpra ranges computed with plain AA and mixed IA/AA are of approximately equal diameter to INTLAB ranges up until around iteration *i* = 750, where the range diameter becomes so small that overhead error begins to dominate. Beyond this point, since internal precision is low and error term trimming is not enabled, the diameters of Arpra ranges are up to 50% larger than those of INTLAB ranges. The small difference between AA and mixed IA/AA results is due to the fact that the plotted diameters are computed from the true_range field of Arpra ranges, and any overhead rounding error present in plain AA ranges is stripped from mixed IA/AA ranges. Besides this, the mixed IA/AA method is only beneficial when transcendental functions are used, since only Chebyshev and Min-Range approximations make direct use of the intersected IA/AA ranges when computing affine terms. In the top right plot of Figure 3, we see that Arpra's mixed trimmed IA/AA begins outperforming INTLAB by a modest amount as the system converges to stability from iteration *i* = 100 onwards, and the range diameter is up to 50% smaller than INTLAB ranges after around iteration *i* = 750, where the rounding errors began dominating in the other methods.

In the bottom row of Figure 3, we see that setting the internal precision higher than the working precision understandably has little effect when the ratio of range diameter to numerical error is high. However, when rounding error becomes significant from around iteration *i* = 750, we see a 30% reduction of range diameter when the internal precision *p* = 54 is one higher than working precision. The remaining plots show that further increases to internal precision have diminishing effects. Increasing precision by a few bits incurs only a small cost in runtime and memory. While this increase in precision is minor compared to an increase from double to quadruple precision (*p* = 53, *p* = 113, respectively), we already see diminishing returns for 3 additional bits, so that further increases in precision are unlikely to make a difference.

Rump's example (Rump, 1988; Loh and Walster, 2002) implies that it is non-trivial to determine how the accuracy of floating-point arithmetic changes as the precision increases, since the mapping from precision to accuracy is not continuous. Despite this, there is clearly a ceiling where the increases in accuracy begin to plateau, suggesting that a more algorithmic way of finding the optimal internal precision is possible. A potential solution, used by the MPFR library (Fousse et al., 2007), is to use Ziv's strategy (Ziv, 1991) as a heuristic. The idea is to start at some base internal precision, just above Arpra's default working precision, and incrementally raise it until the true_range field of an Arpra range is sufficiently tight. A problem with this is that affine ranges are constantly changing, with deviation terms being added, and sometimes removed, and the internal precision would need constant updating. With such a negligible effect on range tightness, such complexity seems of limited use when one can simply set the internal precision moderately high to begin with.

After 500 iterations of the Hénon map, the Arpra ranges representing *x* and *y* each contain approximately 3500 deviation terms, which is enough to cause noticeable slowdown. In order to solve this issue, Arpra implements the deviation term condensing functions discussed in section 2. For a single Arpra range, assuming we are iterating for *m* time steps and that the number of deviation terms grows by some constant *k* each step, we need to compute up to *k* + 2*k* + … + (*m* − 1)*k* + *mk* terms throughout the simulation. Ignoring constants, this gives us an asymptotic runtime complexity of $k\overset{m}{\underset{i=1}{\sum }}i=k\frac{m\left(m+1\right)}{2}=O\left(km^{2}\right)$, which is not ideal in longer computations. Calling arpra_reduce_last_n after each iteration condenses all *k* new (independent) deviation terms into one. Calling arpra_reduce_small_rel condenses all deviation terms smaller than or equal to *tr*, with *t* being the relative threshold and *r* being the range's radius. No more than 1/*t* (rounded down) terms can remain after this call, since the absolute sum of the remaining high-magnitude terms cannot exceed *r*. This effectively resets the number of active noise symbols in a range to some threshold-dependent baseline each time it is used.

**Figure 4:**
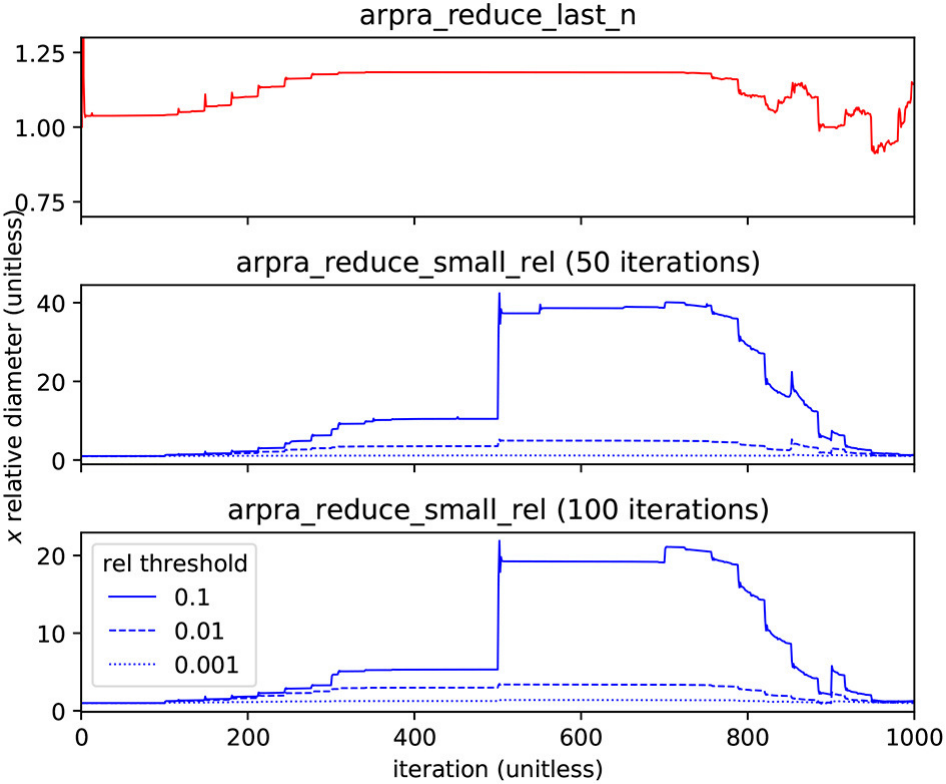
Diameter of the Hénon map's *x* range with term reduction, relative to that of *x* computed without. The top plot shows *x* condensed with arpra_reduce_last_n (red) each iteration, resulting in a reduced deviation term count (see Table 1) at the expense of minor overhead error. The middle and bottom plots show *x* condensed with arpra_reduce_small_rel (blue) every 50 and 100 iterations, respectively, each with relative thresholds 0.001 (dotted), 0.01 (dashed) and 0.1 (solid). Deviation term count is reduced considerably, but range diameters are comparatively larger. Note how the condensing of small deviation terms typically leads to an uptick of the relative diameter every 50 (every 100) steps. The relative threshold of 0.1 is clearly too aggressive, as can be seen by the large jump in relative diameter at step 500.

To illustrate the effectiveness of these functions, we calculated the Hénon map for 1000 iterations using various term reduction strategies, with α = 1.057 and internal precision set to 256. The diameters of term condensed Hénon map *x* ranges, relative to the diameter without term reduction, are shown in Figure 4. For the first term reduction strategy, the arpra_reduce_last_n function is used to condense all new deviation terms in the Hénon map variables after each iteration. This is safe to do because the new noise symbols in *x* and *y* are mutually exclusive. Although it is considered lossless, this function can still introduce overhead rounding error. From the top plot of Figure 4, we can see that arpra_reduce_last_n has a small overhead cost, in terms of diameter growth, but no correlation information is lost and the computational performance gains are significant. A comparison of Arpra term condensing functions is given in Table 1. Despite the performance improvements due to arpra_reduce_last_n, the number of deviation terms in *x* and *y* still grows by one with each iteration, and the computation will still eventually become slow.

**Table 1:** Performance comparison of deviation term condensing functions.

**Method**	**Run time**	**x Terms**	**y Terms**	**Mallocs**	**Malloc bytes**
none	11 min 38 sec	7,005	7,000	146, 727,726	8, 459, 253, 260
last *n*	35 sec	1,002	1,002	23, 653, 899	1, 399, 553, 468
small (0.001*r*)	4.3 sec	126	125	9, 991, 398	569, 101, 594
small (0.01*r*)	2.8 sec	25	26	8, 185, 733	466,901,545
small (0.1*r*)	2.4 sec	2	2	7,745,798	442, 149, 080

*The Hénon map is iterated n = 1,000 times, and the arpra_reduce_small_rel condense epoch is 50 iterations. Term counts are taken at the end. Heap memory information was obtained using Valgrind (Nethercote and Seward, 2007)*.

For the remaining term reduction strategies, the arpra_reduce_small_rel function was used with radius-relative thresholds of 0.001, 0.01 and 0.1. In the middle and bottom plots of Figure 4, the Hénon map variables were condensed every 50 and 100 iterations, respectively. arpra_reduce_small_abs allows finer control of term condensing, however here it makes sense to condense terms that are most weakly contributing to the radius. Due to loss of correlation information, these ranges grow comparatively wider than those condensed with arpra_reduce_last_n. From the plots, we see that ranges grow ever wider as arpra_reduce_small_rel is used more frequently, or with a higher threshold. Indeed, condensing ranges in each iteration or using a high threshold will cause rapid and fatal range growth, in a manner not dissimilar to IA range explosion. However, the number of active deviation terms is far lower with this strategy, as shown in Table 1. From this data, we can see that the majority of deviation terms in both Hénon map ranges have magnitudes <10% of their radius. We also see that careful use of arpra_reduce_small_rel to periodically remove these lesser deviation terms can greatly improve the performance of the analysis, while mindful that excessive use deteriorates range quality. Therefore, some combination of all term reduction strategies seems desirable, where independent terms are condensed as they appear and small terms are swept away when appropriate. Fewer terms translates to less memory and faster processing, and the time and memory savings due to deviation term reduction dwarfs the additional runtime complexity of using these procedures. Conversely, if no reduction procedure is used, Hénon map iterations become noticeably slower as deviation term lists grow unwieldy.

In summary, we found that AA performs well when analysing stable systems, but its usefulness is limited for chaotic systems. It is worth noting, however, that other range analysis methods would also struggle to bound such chaotic computations. We furthermore found that mixed trimmed IA/AA outperforms mixed IA/AA when overhead error dominates range width, and that using higher internal precision helps when rounding error dominates range width. Finally, we tested the effectiveness of Arpra's deviation term condensing functions, finding that overuse of the arpra_reduce_small functions rapidly deteriorates range quality, while carefully combining term reduction strategies significantly improves computational performance.

### 3.3. Spiking Neural Networks

Spiking neural network (SNN) models are used by computational neuroscientists to simulate anything from tiny peripheral neural circuits to vast ‘*in silico*’ brain circuits. Furthermore, there is growing interest in the field of neuromorphic computing (Furber et al., 2014; Diamond et al., 2016a,b). The widespread adoption of SNN simulations has prompted interest in the verification of the resulting data, including data computed on high performance parallel CPU and GPU computing clusters, for instance with tools such as GeNN (GPU enhanced Neural Networks) (Yavuz et al., 2016). Range analysis is particularly important when concurrency is used due to the relationship of rounding errors, resulting non-associativity of numerical operations and the lack of guarantees for the execution order on parallel systems, which can lead to serious issues for replicability, as explained above. It can be an important tool for deciding whether disparate results from two simulation runs are within a range explainable by numerical error or are outside it indicating an algorithmic or programming error.

To study parallel models, we implement equivalent serial models using Arpra. To emulate parallel input current summation, we implemented a function which sums the centre and deviation terms of *n* Arpra ranges with arbitrary summand ordering, using MPFR's correctly rounded mpfr_sum function, and then widens the resulting range by the rounding error bound for recursive summation. A tight rounding error bound for summation is given in Rump (2012).

(14) $$\begin{array}{l} |\tilde{S}-S|\leq \left(n-1\right)\text{u}\overset{n}{\underset{i=1}{\sum }}|x_{i}|. \end{array}$$

where *S* is the exact sum, $\tilde{S}$ is the result of summing with *n* − 1 correctly rounded floating-point additions, and *x* is the vector of summands. When computing this error bound with Arpra ranges, the absolute value of a summand range is defined as the true_range bound with the highest magnitude. This error is accumulated with other rounding errors into the new deviation term.

There are many different neuron and synapse models that are used in SNN simulations with varying degrees of abstraction. Some of the more popular models include the Izhikevich neuron model (Izhikevich, 2003) and the Traub-Miles type (Traub and Miles, 1991) Hodgkin-Huxley neuron model. In these experiments, we use the Morris-Lecar neuron model (Morris and Lecar, 1981), a reduced version of a Hodgkin-Huxley conductance based model, and the Traub-Miles neuron model. Furthermore, we use a variant of the Rall synapse model (Rall, 1967) which has been modified to remove discontinuities. All models used in this study are fully continuous (see section 2). If hybrid systems such as the popular integrate-and-fire neuron or the Izhikevich neuron model (Izhikevich, 2003) were to be used, their discretised spiking dynamics (instantaneous spike detection and voltage reset) can cause simulation trajectories to be partitioned into two or more regions. This would require the capability to split affine ranges into smaller sub-ranges, and the ability to merge these ranges as and when the trajectories converge again. This is non-trivial, since modifying affine ranges can invalidate correlation information. The Arpra library does not currently support this, and we will hence use the continuous models throughout our analysis. For the topology of the network we chose a simple fan-in SNN model, in which multiple Poisson neuron inputs project to a single neuron via modified Rall synapses.

In all tests, the working precision of Arpra ranges was 53 bits, equivalent to IEEE-754 double-precision, while Arpra's internal precision was set to 256 bits. All models were integrated with a forward Euler algorithm, in steps of *h* = 0.5 ms for Morris-Lecar models and *h* = 0.05 ms for Traub-Miles models. Arpra transcendental functions used the Chebyshev approximation scheme. Independent deviation terms were merged each iteration with arpra_reduce_last_n, and terms ≤10% of the radius were merged every 100 iteration with arpra_reduce_small_rel.

**Figure 5:**
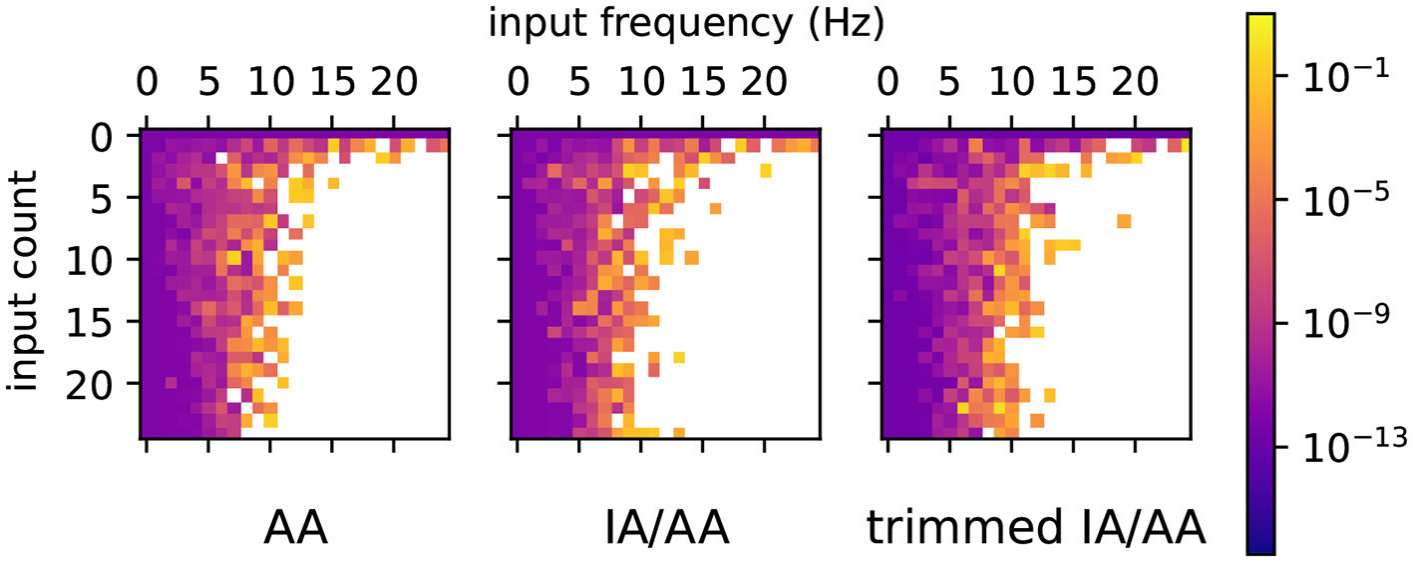
Simulations of fan-in Morris-Lecar networks where each model had a different number of Poisson input neurons (0–24 neurons) and the inputs had different frequencies (0–24 Hz). Each tile shows the mean diameter of *V* over 500 simulated milliseconds, with warmer tiles indicating wider mean diameter. White tiles indicate that the range explodes at some point during the simulation.

We first tested how changes in input count and spike frequency affect range growth. A number of fan-in Morris-Lecar networks were simulated for 500 ms, where each model had a different number of Poisson input neurons and a different input firing rate, varied between 0 and 24 inclusive. Random number seeds for Poisson input generators and synaptic conductance values are *not* fixed in this experiment. The average diameter of the *V* range is shown for all three Arpra methods in Figure 5. White tiles indicate that a range exploded within the 500 ms of simulated time. Otherwise, warmer tiles indicate a higher average range width. Our results indicate that both increasing the number of Poisson input neurons and increasing the spiking frequency of the inputs affects the average diameter of ranges over the course of the simulation. In these simulations, ranges tend to begin exploding when input neuron spiking frequency exceeds around 10 Hz. How the range depends on parameters such as the firing rate and number of input neurons depends on a number of factors but essentially on how much the parameter affects the stability of the system. For instance, changing the firing rate of a large number of input neurons is likely to have a more potent effect than adding a single input neuron.

**Figure 6:**
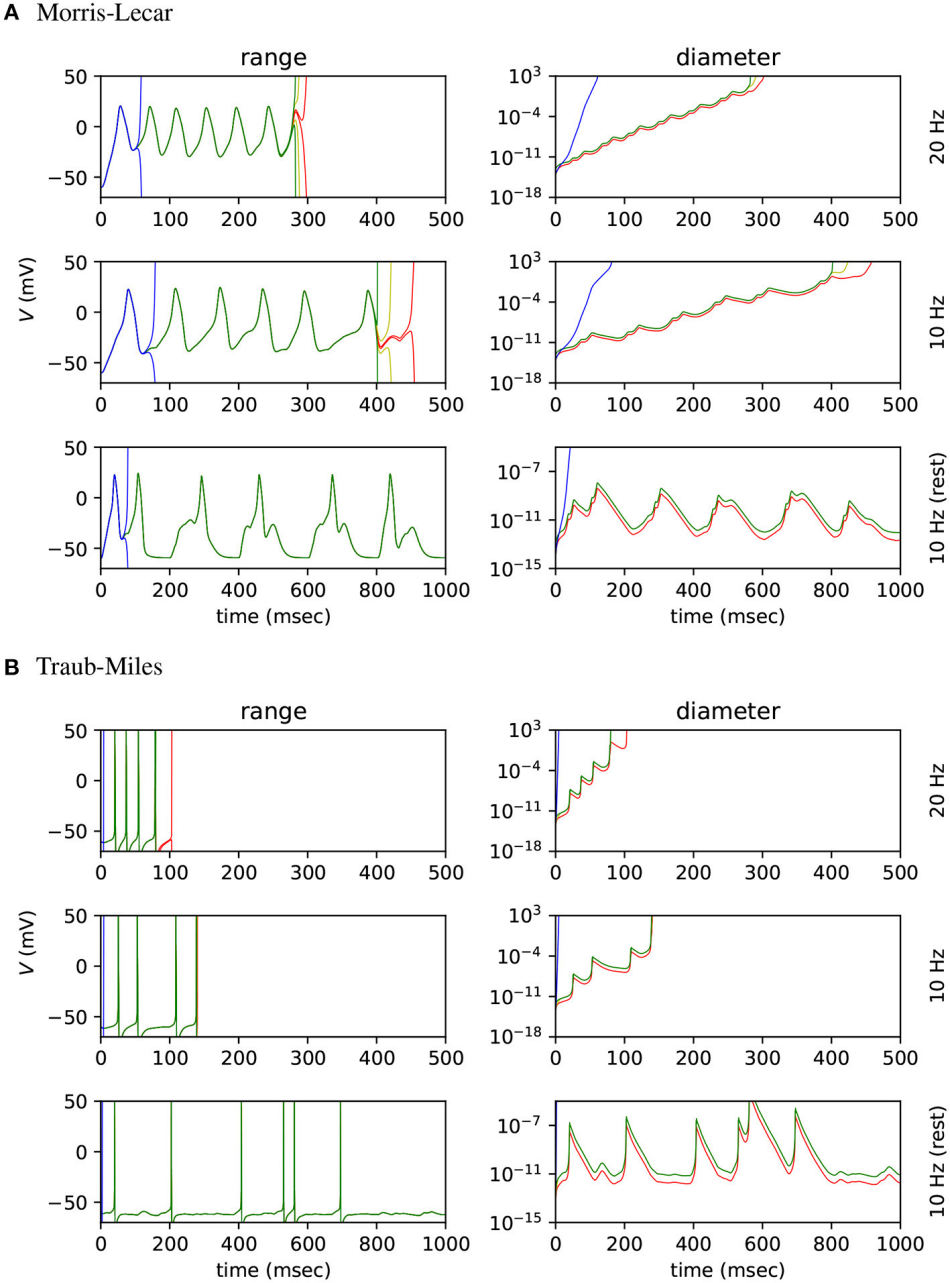
Range and diameter of *V* in a fan in SNN model, where 50 Poisson input neurons project to a single output neuron. **(A)** Results for the Morris-Lecar model. **(B)** results for the Hodgkin-Huxley type Traub-Miles model. The three sub-panels show, respectively, results for constant input firing rate λ = 20 Hz, λ = 10 Hz, and for alternating 100 ms bursts of λ = 10 Hz input and 100 ms of λ = 0 Hz rest. Arpra methods AA (green), mixed IA/AA (yellow) and mixed Trimmed IA/AA (red) are compared to the MPFI (Revol and Rouillier, 2005) implementation of IA (blue).

We then tested how input spike frequency affects range growth in each range analysis method. Fifty Poisson input neurons were used to stimulate Morris-Lecar and Traub-Miles neurons in fan-in networks. The random number generator seed used to generate spikes and initialise synaptic conductances was fixed for all tests. Plot A of Figure 6 shows the results for the Morris-Lecar model simulations, while plot B shows the results for the Traub-Miles model simulations. The top row of each plot shows the range and diameter of the output neuron's membrane potential *V*, with input firing frequency λ = 20 Hz, and the second row shows the range and diameter of *V* with λ = 10 Hz. For the third row of Figure 6, input spike frequency is alternated between 100 ms bursts of λ = 10 Hz input and 100 ms λ = 0 Hz rest periods.

Note how IA ranges explode almost immediately in all experiments. The affine Arpra ranges last considerably longer, however they too eventually explode when the model is subject to sustained instability. The diameter difference of ranges computed by the three Arpra methods is noticeable, but only just. The mixed trimmed IA/AA method slightly outperforms other AA methods, however this difference is small since the majority of range growth occurs due to system instability. For the same reason, increasing either the internal or working precision of Arpra ranges also has little effect on range diameter. Whilst these results are not what one might have hoped for, one might argue that this is to be expected. The experiments of section 3.2 showed us that even AA ranges explode if a system is sufficiently unstable. One might expect ranges of all methods to grow slower in more stable systems, just as the stable Hénon map ranges did in section 3.2. The top two rows in Figure 6A show that this is indeed still the case, with ranges in the simulation with λ = 10 Hz inputs lasting approximately 200 simulated milliseconds longer than those of the simulation with λ = 20 Hz inputs in the Morris-Lecar experiment. A similar effect is shown for the Traub-Miles model in Figure 6B.

The question then becomes whether or not Arpra ranges can still recover once the SNN simulation enters a stable system regime, after a period of growth in an unstable regime. The bottom rows of Figures 6A,B illustrate that, although the diameter of *V* grows rapidly during the spike burst regime, it also shrinks equally rapidly in the quiescent regime to a baseline of approximately 10^−13^ for the Morris-Lecar model, and approximately 10^−12^ for the Traub-Miles model. This is consistent with the behaviour of Arpra when iterating the stable Hénon map in section 3.2, where range width begins to shrink once the stable limit cycle is reached. Although this demonstrates that AA at least has the ability to recover from moderate range explosion in chaotic regimes, the other results in this section suggest that the scope of all three AA variants discussed here may be limited to the analysis of SNN models with relatively low spiking activity. It is clear that dynamical systems simulation trajectories must have local stability for a sufficiently high proportion of the simulation to be amenable for analysis using any AA method.

**Figure 7:**
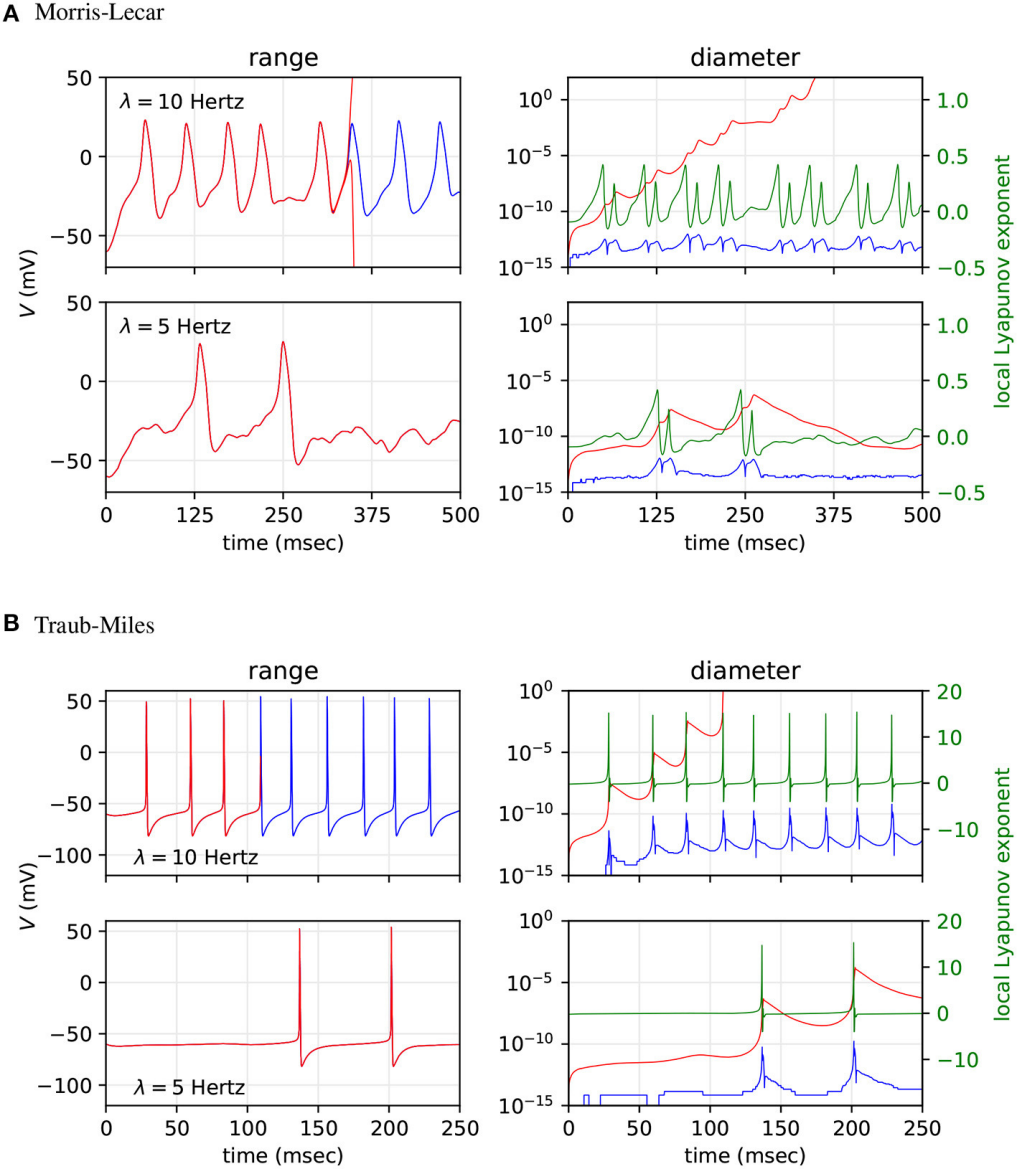
Comparison of mixed trimmed IA/AA ranges (red) and the trajectory boundaries computed from 1, 000 floating-point simulations (blue) of a fan-in network with *n* = 500 Poisson input neurons. **(A)** Results for the Morris-Lecar model. **(B)** Result for the Hodgkin-Huxley type Traub-Miles model. Top sub-panels show results with input spike frequency λ = 10 Hz and bottom sub-panels with λ = 5 Hz. We analysed the range of *V* (left sub-panels), and the diameter of these ranges (right sub-panels). For context, the local Lyapunov exponent is plotted in the right sub-panels (green).

Next, we asked how the worst case ranges actually compare to the typically observed variability due to unpredictable summation orders in simulations using IEEE-754 floating-point arithmetic on parallel hardware. To test how well Arpra bounds the trajectories of floating-point SNN simulations, we analysed fan-in Morris-Lecar and Traub-Miles networks with *n* = 500 Poisson input neurons with λ = 10 Hz and λ = 5 Hz firing rate using Arpra's mixed trimmed IA/AA method. All random number seeds were fixed. We then simulated the same models 1000 times with standard IEEE-754 floating-point arithmetic using the MPFR library (Fousse et al., 2007) and with randomised incoming spike lists, to simulate the unpredictable summation order of input currents on parallel hardware. We then compared the observed upper and lower bounds of the 1, 000 sampled floating-point trajectories with the ranges computed by Arpra. The ‘diameter’ floating-point trajectories at a given time is defined as the difference between the maximum and minimum trajectory value at that time, while the diameter of an Arpra range is defined as the range's true_range field. Furthermore, we performed a stability analysis on the networks to determine how the growth of Arpra ranges and the divergence of observed trajectories in the floating-point computations relate to the stability of the system. We used the tangent space method to calculate the largest local Lyapunov exponent as a function of time. The results are plotted in Figure 7.

As the top rows of A and B in Figure 7 show, the diameter of ranges computed with mixed trimmed IA/AA grows quickly towards infinity with λ = 10 Hz model input, whilst the divergence of observed trajectories computed in floating-point remains relatively constant throughout, diverging slightly when spikes occur but converging back afterwards. In section 3.2, we saw that affine ranges inevitably explode when analysing chaotic systems, and this is reflected in these results. The global Lyapunov exponent of the floating-point simulations' average trajectory is 0.041 to three decimal places on the Morris-Lecar model, and 0.117 on the Traub-Miles model, indicating that these trajectories are indeed overall unstable. We see that, although the Arpra ranges have brief recovery periods when the local Lyapunov exponent falls below zero due to the absence of spiking dynamics, the ranges resume growing when the local Lyapunov resurfaces above zero. We also see that the shape of neuronal spikes has a notable effect on the overall stability of the trajectory, and range diameter growth is consequently much higher during Traub-Miles spikes than in Morris-Lecar spikes.

Since we already know that affine ranges explode in chaotic systems, it is perhaps more interesting to ask how tight the bounds are compared to the variation of floating-point trajectories in a more stable system. In the bottom row of plot A in Figure 7, we see that the diameter of the *V* range is allowed to recover fully down to a baseline value in the absence of Morris-Lecar spiking dynamics. Similarly, in plot B, we see the diameter of *V* rapidly recovers in the absence of Traub-Miles spiking dynamics. However, the baseline diameter of *V* is still approximately three orders of magnitude higher, at least, than the range of observed divergence of trajectories in the repeated floating-point simulations. Global Lyapunov exponents of −0.015 to three decimal places on the Morris-Lecar model, and −0.013 on the Traub-Miles model, confirms that these trajectories are overall stable, and local Lyapunov exponents converge below zero after the Arpra range value bottoms out. So what could be the cause of this additional range width?

First and foremost, it is important to remind ourselves that range analysis, by its very definition, is a method for computing the theoretical worst case error bounds of a computation, and not necessarily the bounds that one may observe in practice. Range analysis is conservative by design. Having said that, there are different flavours of range analysis. We have already seen how much of an improvement AA is over IA, but AA is a first-order range analysis method, and thus incurs heavy approximation error whenever nonlinear functions are used. One solution, and potential future work in Arpra, is to implement Taylor intervals, in which ranges are represented using Taylor series polynomials. This would likely be of most benefit for moderately stable nonlinear computations. For now, however, we focus on the linear mixed trimmed IA/AA method.

**Figure 8:**
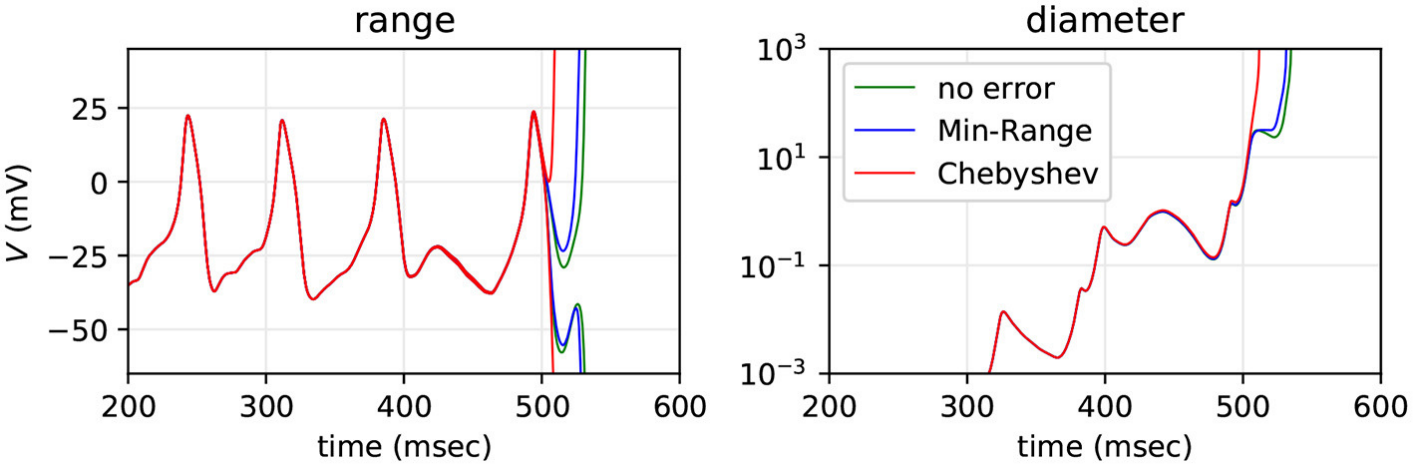
Range and diameter of the Morris-Lecar neuron's *V* variable, computed with transcendental functions using Chebyshev approximation (red), Min-Range (blue) and Chebyshev with approximation error set to zero (green). The model is a fan-in SNN with *n* = 50 Poisson inputs firing at λ = 10 Hz.

The Morris-Lecar neuron model in Equation (4) uses the nonlinear functions tanh and cosh, implemented in terms of arpra_exp, and division, implemented using arpra_inv. The Traub-miles model in Equation (6) is also implemented using arpra_exp and arpra_inv. These functions are both susceptible to overshoot and undershoot, as discussed in section 1. Because of this, one would expect there to be a noticeable difference in the diameter of Arpra ranges when different approximation schemes are used. In order to determine how the error from nonlinear function approximation affects Arpra's mixed trimmed IA/AA ranges in SNN models, we simulated a fan-in Morris-Lecar network with 50 Poisson input neurons with λ = 10 Hz firing rate three times. In the first run, arpra_exp and arpra_inv use the same Chebyshev approximation scheme used up until now. In the second run, these functions use the Min-Range approximation scheme. In the final run, functions use the Chebyshev scheme, but the approximation error term δ is set to zero, giving us a crude demonstration of the effect linearisation error has on computed ranges. Random number seeds are fixed. The results are shown in Figure 8.

We see that the Min-Range function approximation scheme performs only marginally better than the Chebyshev scheme, with ranges lasting approximately 20 simulated milliseconds longer before exploding. This extra accuracy can be attributed to the lack of overshoot and undershoot in the Min-Range approximation. When artificially removing the approximation error altogether, we see that ranges still barely last longer than ranges with approximation error added. One can imagine that a second order range analysis method would better approximate the exp function using a quadratic curve, lowering the error term δ, and successively higher order methods would further reduce δ. Our results suggest, however, that system instability is by far the biggest contributor to catastrophic range growth, which in turn suggests that this growth is an appropriate estimate of the worst case. But in practice this worst case appears to not be realised, due to cancellation of errors, as seen in the empirical test with 1, 000 repeated floating-point simulations.

## 4. Discussion

The original motivation that culminated in this work was to compute boundaries for the numerical error of GeNN (Yavuz et al., 2016) simulations of spiking neural networks (SNN) on massively parallel hardware. In the pursuit of this goal, the Arpra library for arbitrary-precision range analysis was developed. Unlike other AA packages, Arpra builds on the standard AA method by exploiting extended precision floating-point arithmetic using the MPFR library (Fousse et al., 2007) to produce tighter bounding ranges. It also features the novel mixed trimmed IA/AA method and three novel term reduction functions to further decrease overhead error and improve computational tractability.

We analysed the two-dimensional Hénon map (Hénon, 1976) and a fan-in SNN model involving Morris-Lecar neurons (Morris and Lecar, 1981) and modified Rall synapses (Rall, 1967). We found that the mixed trimmed IA/AA method and extended internal precision are most advantageous when the ratio of overhead error to range diameter is high, but are less significant when the converse is true, and when the computation is too unstable for ranges to recover. When using Arpra's deviation term condensing strategies, a small overhead rounding error cost was demonstrated when using the lossless arpra_reduce_last_n routine in the Hénon map problem. However, the benefits were an approximate 85% reduction of deviation terms and a large decrease in both the runtime and memory usage. We saw even more aggressive reduction of deviation terms using the lossy arpra_reduce_small_rel routine, with an over 99% reduction of deviation terms using the relative threshold 0.1. However, due to the loss of correlation information, it was found that this routine should be used sparingly to avoid catastrophic range growth, and is most effective when used in combination with other term reduction strategies.

Here, we feel compelled to repeat that, while the computed ranges can appear loose compared to the variability of plain floating-point computations observed in practice, the Arpra library is behaving correctly. Arpra obeys the ‘fundamental theorem of interval arithmetic’. That is to say that the range computed by arithmetic operations must always contain the result for every single point inside the operand ranges. It always computes the worst-possible-case error boundaries of all computations, no matter how extreme. While, rounding errors are certainly not random, it stands to reason that floating-point rounding errors incurred using IEEE-754 ‘round to nearest’ mode are a somewhat even mixture of positive and negative errors. If one were to make the simplifying assumptions that rounding errors are uniformly distributed in some constant interval [−*k, k*], and these rounding errors are independent, then the central limit theorem states that a floating-point number containing *n* rounding errors should be normally distributed about a mean somewhere near the centre of the corresponding Arpra range, with a standard deviation of $k\sqrt{n}$, which can be much smaller than a worst case error of *k* · *n*.

Arpra can still be a useful tool for examining the short-term trajectory divergence and identifying points of instability in chaotic systems. One could simply begin analysis at or slightly before points of specific interest. Although Arpra performs well in most linear computations, and reasonably well in sufficiently stable nonlinear computations, its performance begins to decline as the nonlinear dynamics begin to dominate the computation. Since the AA method consists of linear functions of the centre and deviation terms, the logical progression for range analysis would be to allow higher-order terms in the range representations. For instance, one might approximate the exponential function with *n*th-order deviation terms, with *n* + 1 order approximation error. These ideas were proposed in Berz and Hoffstätter (1998) and Berz and Makino (1998), under the name ‘Taylor methods’. They were subsequently used to successfully model near-Earth object trajectories in space, given intervals of initial conditions (Berz et al., 2001). We leave the implementation of Taylor method range analysis for future work.

Comprehensive analysis packages like the Astrée static analysis package (Cousot et al., 2007) are often proprietary and expensive, which rules out their use in the analysis of open source numerical software. Arpra (Turner, 2019), on the other hand, is open source and freely available under the terms of the GNU lesser general public license version 3.0. Arpra also has the advantage of being built on top of the arbitrary-precision MPFR library (Fousse et al., 2007), and benefits from arbitrary floating-point precision and correct rounding for all arithmetic functions in all software and hardware environments. Stolfi and de Figueiredo (2007) give plenty of examples where the AA method is useful, such as function root finding and global optimisation. Arpra has many more uses besides these, such as for the verification of numerical libraries, both proprietary and otherwise. Open source libraries can lack tight accuracy bounds for functions in their documentation. Given the improved performance of Arpra in linear computations, the analysis of software like glibc and many linear algebra packages could also be prime use cases for Arpra.

## Data Availability Statement

The Arpra software is available at https://github.com/arpra-project/arpra. Scripts to generate the data underlying the figures are included at https://github.com/arpra-project/arpra/tree/master/extra.

## Author Contributions

JT developed Arpra, ran the numerical experiments and made the figures. JT and TN wrote the manuscript and revised it. Both authors contributed to the article and approved the submitted version.

## Conflict of Interest

The authors declare that the research was conducted in the absence of any commercial or financial relationships that could be construed as a potential conflict of interest.

## Figures and Tables

**Figure A1:**
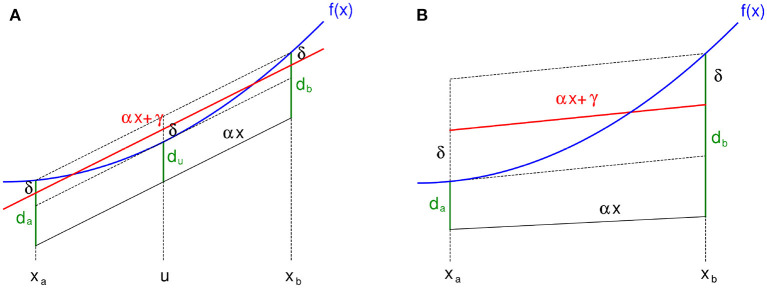
Illustration of the two function approximations used in Arpra. **(A)** Chebyshev approximation and **(B)** Min-Range approximation. Note how by construction, the maximal errors δ are incurred at the end points *x*_*a*_ and *x*_*b*_ of the range in both approximations.

## Appendix

The following are proofs that the mix_trim procedure in Algorithm 1 maintains correctness of ranges computed by affine Chebyshev and Min-Range approximations of a univariate function $ŷ=f\left(\hat{x}\right)$, so long as the second derivative of *f* does not change sign within the input range (i.e., *f* is convex/concave on $\hat{x}$). The proofs are largely based on the ideas of Stolfi and de Figueiredo (2007).

### A. Chebyshev Approximation

Let *f*:[*x*_*a*_, *x*_*b*_] → ℝ be some twice differentiable real-valued function on the interval $\left[x_{a},x_{b}\right]=\hat{x}.t$, which second derivative does not change sign within [*x*_*a*_, *x*_*b*_]. We assume infinite floating-point precision in this proof.

Let $\alpha =\frac{f\left(x_{b}\right)-f\left(x_{a}\right)}{x_{b}-x_{a}}$ be the slope of the the linear Chebyshev approximation of *f*, and let *d*_*a*_ = *f*(*x*_*a*_) − α*x*_*a*_, *d*_*b*_ = *f*(*x*_*b*_) − α*x*_*b*_, and *d*_*u*_ = *f*(*u*) − α*u*, where *u* ∈ [*x*_*a*_, *x*_*b*_] is a point where the first derivative of *f* is equal to the slope α of the approximation - i.e., *f*′(*u*) = α. The existence of *u* is guaranteed by the mean value theorem. The choice of α ensures that *d*_*a*_ = *d*_*b*_.

Let $\gamma =\frac{d_{a}+d_{u}}{2}$ and $\delta =\frac{\left|d_{a}-d_{u}\right|}{2}$. The Chebyshev approximation then is given as α*x* + γ.

For definiteness and without loss of generality we assume *f* is convex on [*x*_*a*_, *x*_*b*_] (if it is concave, use −*f* to find a similar result). Then, because *f* is convex, *f*(*x*) ≥ α*x* + *d*_*u*_ for all *x* ∈ [*x*_*a*_, *x*_*b*_], the distance to the tangent α*x* + *d*_*u*_ is increasing monotonically to the left and the right of *u*, and hence, by construction, the maximum errors above and below the linear approximation are equal, and occur at, respectively, *x*_*a*_ (and *x*_*b*_) and *u* (see Figure A1A):

(A1) $$\begin{array}{l} \max\left\{f\left(x\right)-\left(\alpha x+\gamma \right):x\in \left[x_{a},x_{b}\right]\right\}=f\left(x_{a}\right)-\left(\alpha x_{a}+\gamma \right)\\ \;=d_{a}-\frac{d_{a}+d_{u}}{2}\\ \;=\frac{d_{a}-d_{u}}{2}=\delta ,\text{and} \end{array}$$

(A2) $$\begin{array}{l} \max\left\{\alpha x+\gamma -f\left(x\right):x\in \left[x_{a},x_{b}\right]\right\}=\alpha u+\gamma -f\left(u\right)\\ \;=-d_{u}+\frac{d_{a}+d_{u}}{2}\\ \;=\frac{d_{a}-d_{u}}{2}=\delta . \end{array}$$

Therefore, δ is the amplitude for the new deviation coefficient. Let $ŷ.c=\alpha \hat{x}.c+\gamma$, $ŷ.d\left[i\right]=\alpha \hat{x}.d\left[i\right]$ for *i* < ŷ.*n* and ŷ.*d*[*n*] = δ, and let $ŷ.r=\sum_{i}|ŷ.d\left[i\right]|$, as per standard AA. Intersecting AA and IA ranges, we have

(A3) $$\begin{array}{l} ŷ.t=T\cap f\left(\left[x_{a},x_{b}\right]\right),\text{where}T=\left[\left(ŷ.c-ŷ.r\right),\left(ŷ.c+ŷ.r\right)\right]. \end{array}$$

The mixed trimmed method then trims ŷ.*d*[ŷ.*n*] by *w* = min{*w*_lo_, *w*_hi_}, where *w*_lo_ = ŷ.*t*.*lo* − *T*.*lo* and *w*_hi_ = *T*.*hi* − ŷ.*t*.*hi*. If *f*(*x*_*b*_) ≥ *f*(*x*_*a*_) then α ≥ 0 and we find

(A4) $$\begin{array}{l} w_{\text{hi}}=\alpha \hat{x}.c+\gamma +\alpha \hat{x}.r+\delta -f\left(x_{b}\right)=\alpha x_{b}+\gamma -f\left(x_{b}\right)+\delta =0, \end{array}$$

and if *f*(*x*_*b*_) < *f*(*x*_*a*_) then α < 0 and we find

(A5) $$\begin{array}{l} w_{\text{hi}}=\alpha \hat{x}.c+\gamma +|\alpha \hat{x}.r|+\delta -f\left(x_{a}\right)\\ \;=\alpha x_{a}+\gamma -f\left(x_{a}\right)+\delta =0 \end{array}$$

as well. Because the IA interval is always smaller than *T*, *w*_lo_ and *w*_hi_ are bounded by 0 from below, and hence (4) and (5) imply *w* = 0 (except for rounding errors not treated here because we assumed infinite precision). Because the trim *w* is 0, the range is guaranteed not to be invalidated by trimming.

### B. Min-Range

Let, again, *f*:[*x*_*a*_, *x*_*b*_] → ℝ be twice differentiable with second derivative that does not change sign within [*x*_*a*_, *x*_*b*_] and assume infinite floating-point precision.

If *f* is non-monotonic, then the Min-Range approximation is equivalent to the IA interval, which means the trim is automatically 0 and the AA range will not be compromised by trimming.

If *f* is monotonic, let $u=\mathrm{argmin}\left\{\left|f^{\prime }\left(x_{a}\right)|,|f^{\prime }\left(x_{b}\right)\right|\right\}$ and α = *f*′(*u*). Define *d*_*a*_ = *f*(*x*_*a*_)−α*x*_*a*_ and *d*_*b*_ = *f*(*x*_*b*_)−α*x*_*b*_ and let $\gamma =\frac{d_{a}+d_{b}}{2}$ be the midway point between *d*_*a*_ and *d*_*b*_. Let $\delta =\frac{\left|d_{a}-d_{b}\right|}{2}$ be half the distance from *d*_*a*_ to *d*_*b*_. The Min-Range approximation is then given as α*x* + γ.

Assume for definiteness that *f* is convex in [*x*_*a*_, *x*_*b*_] and α ≥ 0, and hence *u* = *x*_*a*_, see Figure A1B (all other combinations of convex or concave, α ≥ 0 or α < 0 work in a similar way). Because *f* is convex and *u* = *x*_*a*_, *f*(*x*) ≥ α*x* + *d*_*a*_ on [*x*_*a*_, *x*_*b*_] and the distance of *f* to the tangent α*x* + *d*_*a*_ increases monotonically for increasing *x*. Hence, by construction, the maximum errors below and above the linear approximation are equal, and occur at, respectively, *x*_*a*_ and *x*_*b*_ (Figure A1B):

(A6) $$\begin{array}{l} \max\left\{\alpha x+\gamma -f\left(x\right):x\in \left[x_{a},x_{b}\right]\right\}=\alpha x_{a}+\gamma -f\left(x_{a}\right)\\ \;=-d_{a}+\frac{d_{a}+d_{b}}{2}\\ \;=\frac{d_{b}-d_{a}}{2}=\delta ,\text{and} \end{array}$$

(A7) $$\begin{array}{l} \max\left\{f\left(x\right)-\left(\alpha x+\gamma \right):x\in \left[x_{a},x_{b}\right]\right\}=f\left(x_{b}\right)-\left(\alpha x_{b}+\gamma \right)\\ \;=d_{b}-\frac{d_{a}+d_{b}}{2}\\ \;=\frac{d_{b}-d_{a}}{2}=\delta . \end{array}$$

Therefore, δ is the amplitude for the new deviation coefficient. Let $ŷ.c=\alpha \hat{x}.c+\gamma$, $ŷ.d\left[i\right]=\alpha \hat{x}.d\left[i\right]$ for *i* < ŷ.*n* and ŷ.*d*[*n*] = δ, and let $ŷ.r=\sum_{i}|ŷ.d\left[i\right]|$, as per standard AA. Intersecting AA and IA ranges, we then have

(A8) $$\begin{array}{l} ŷ.t=T\cap f\left(\left[x_{a},x_{b}\right]\right),\text{where}T=\left[\left(ŷ.c-ŷ.r\right),\left(ŷ.c+ŷ.r\right)\right]. \end{array}$$

The mixed trimmed method then trims ŷ.*d*[ŷ.*n*] by *w* = min{*w*_lo_, *w*_hi_}, where *w*_lo_ = ŷ.*t*.*lo*−*T*.*lo* and *w*_hi_ = *T*.*hi*−ŷ.*t*.*hi*. This is

(A9) $$\begin{array}{l} w_{\text{lo}}=f\left(x_{a}\right)-\left(\alpha \hat{x}.c+\gamma -\alpha \hat{x}.r-\delta \right)\\ \;=f\left(x_{a}\right)-\left(\alpha x_{a}+\gamma \right)+\delta =0,\text{and} \end{array}$$

(A10) $$\begin{array}{l} w_{\text{hi}}=\alpha \hat{x}.c+\gamma +\alpha \hat{x}.r+\delta -f\left(x_{b}\right)=\alpha x_{b}+\gamma -f\left(x_{b}\right)+\delta =0. \end{array}$$

So, as for the Chebyshev approximation above, the eventual trim is *w* = 0 (except for rounding errors not treated here because of our assumption of infinite precision).

In summary, both Chebyshev and Min-Range approximations only trim additional rounding errors and leave the correctness of the mixed IA/AA method intact.
